# Spatiotemporal transcriptomic mapping reveals region-specific glial activation and astrocyte shifts in epileptogenesis beyond the hippocampus

**DOI:** 10.1186/s40478-026-02224-y

**Published:** 2026-01-15

**Authors:** Adrien Dufour, Christophe Le Priol, Baptiste Porte, Ronan Jouanard, Julien Maurizio, Anne-Elodie Receveur, Stéphane Auvin, Juliette Van Steenwinckel, Pierre Gressens, Andrée Delahaye-Duriez

**Affiliations:** 1 NeuroDiderot UMR 1141, Université Paris Cité and INSERM, 75019 Paris, France; 2 Inovarion, 75005 Paris, France; 3 Service de Neuropédiatrie, Hôpital Robert Debré, AP-HP, 75019 Paris, France; 4 Faculty of Medicine, UFR SMBH, Université Sorbonne Paris Nord, 93000 Bobigny, France; 5 Unité Fonctionnelle de Médecine Génomique et Genetique Clinique, Hôpital Jean Verdier, AP-HP, 93140 Bondy, France

**Keywords:** Epileptogenesis, Spatial transcriptomics, Temporal lobe epilepsy, Astrogliosis, Microglia, Thalamus, Latent phase

## Abstract

Temporal lobe epilepsy (TLE) is a prevalent neurological disorder often preceded by an initial precipitating event, followed by a latent phase, and culminating in chronic epilepsy with recurrent seizures. The molecular and cellular mechanisms driving this transformation remain incompletely understood. Here, we applied Visium-based spatial transcriptomics to coronal brain sections from lithium-pilocarpine-induced *status epilepticus* (SE) rats and controls (n = 16) to map transcriptional dynamics across epileptogenesis. Spatial clustering accurately defined anatomically relevant regions and canonical markers in controls. Comparative analyses revealed extensive SE-associated transcriptional alterations spanning latent and chronic phases across all examined regions. Notably, spatial profiling demonstrated that microglial activation and reactive astrogliosis extended well beyond the hippocampus, encompassing white matter tracts and multiple thalamic nuclei during the latent phase. Cell-type deconvolution further identified pronounced regional shifts in astrocyte functional subtypes within these reactive zones. These findings uncover the spatial heterogeneity of epileptogenic processes, highlighting previously underappreciated thalamic and white matter involvement. The identification of region-specific glial responses and astrocyte subtype transitions provides new mechanistic insights into epileptogenesis and underscores the need for region- and cell-type–targeted strategies to inform therapeutic interventions in TLE.

**Supplementary Information:**

The online version contains supplementary material available at 10.1186/s40478-026-02224-y.

## Introduction

Epilepsy, in all its forms is one of the most common neurological disorders in both children and adults, with a global prevalence ranging from 4 to 10 per 1000 people and approximately 50 million affected individuals worldwide [68, 79]. Seizures result from abnormal synchronous neuronal activity, either focal or generalized, causing behavioral and cognitive manifestations of varying severity [23]. While genetic mutations and structural brain lesions account for over half of cases, more than one-third remain of unknown etiology [7]. Despite the availability of numerous antiepileptic drugs, approximately 30% of patients develop drug-resistant epilepsy [35]. Temporal lobe epilepsy (TLE) represents ~ 30% of all epilepsies and follows a characteristic progression [54]. An initial precipitating injury (IPI)—such as prolonged febrile seizures, *status epilepticus* (SE), head trauma, or encephalitis—triggers the transformation of a previously neurotypical brain into an epileptic one. This IPI is followed by a variable latent period (months to years) without clinical seizures, before patients enter the chronic phase marked by recurrent seizure clusters [39].

The processes that link brain injury such as IPI or other predisposing factors to the emergence of epilepsy are grouped under the term of epileptogenesis [24, 45]. Epileptogenesis involves pathological changes across several hierarchical levels: molecular, cellular, histological and neural circuit organization. At the molecular level, genes related to excitatory and inhibitory receptors, growth factors such as *VEGF*, *BDNF* or *NGF*, transcription factors, synapse organization and function, immune response and inflammation, and gliosis have their expression altered [4, 26, 56, 57, 77]. At the cellular level, these dysregulations result in alterations in axons, dendrites and/or white matter leading to neuronal death but also axonal and/or synapse sprouting, neurogenesis, glial activation and proliferation. At the histological level, hippocampal sclerosis appears characterized by neuronal loss in CA1, CA2, CA3, and dentate gyrus regions, accompanied by mossy fiber sprouting and astrogliosis [30, 64]. Finally, these damages impact the organization of neural circuits and explain the abnormal positive feedback, responsible for higher excitability and/or abnormal synchronous activation [19, 70].

The classical lithium-pilocarpine rodent model recapitulates electro-pathophysiological features observed in human TLE and was widely used to investigate epileptogenesis and evaluate potential protective effects of drugs [15, 16, 40]. Pilocarpine disrupts the equilibrium between excitatory and inhibitory inputs and induces SE. Acute SE effects can be observed until 3 days after SE [2, 9, 12, 51]. The total duration of the latent period itself varies around 2–4 weeks depending on individuals, SE duration, the method of SE termination, strain, age and gender [15, 76].

In recent years new sequencing technologies called spatial transcriptomics (ST) emerged, allowing the genome-wide profiling of transcripts while preserving their spatial location on histological sections [62, 67]. ST has already proven its efficiency and usefulness notably for neuroscience research [13, 47, 52]. This technology has been rarely applied to study epilepsy. The genome-wide Visium technology was used for a few patients with Focal cortical dysplasia type 2 to accurately describe the transcriptional changes associated with the dysmorphic neurons and balloon cells that characterize this disease [6, 75]. In another animal model of TLE (injection of kainic acid into the CA1 region of the hippocampus of mice) a version of ST limited to a panel of 247 genes was applied with a small number of sections restricting the analysis to one epileptic and one control sections at a single time point [43].

Our study aimed to investigate the temporal and spatial transcriptional dysregulations during epileptogenesis. We collected brains from both rodent lithium-pilocarpine-induced SE rats and lithium-control counterparts to apply the Visium spatial transcriptomics technology.

To conduct a comprehensive study of processes happening during the latent and the chronic phases of this model of TLE we selected four time points (5, 10, 20 and 40 days after SE). We identified spatially defined clusters and subclusters overlapping anatomical brain regions and performed differential expression and differential pathway activity analyses between brains from pilocarpine-induced SE rats and control (CRTL) animals at the four time points. Pathway and cell-type annotation were also analyzed for each spot and visualized on brain sections. We identified SE induced microglial activation and reactive astrogliosis in the hippocampus, white matter and localized parts of the thalamus during the whole latent phase.

## Material and methods

### Rodent model of temporal lobe epilepsy

Thirty young Wistar Hanover male rats (3–4 weeks old and 101–125 g) were obtained from Charles River Laboratory (Saint Germain Nuelles, France). Animals were housed in standard cages by pair in a controlled environment (20–24 °C, 45–65% humidity, and 12 h/12 h light cycle). Rats had ad libitum access to food and water. The experimental protocol was approved by the ethical committee for animal experimentation of Robert Debré Hospital (Paris, France) and by the Committee of the Higher Education, Research and Innovation French Ministry (agreement APAFIS #22456-2017121923468530 v3). All experiments followed animal handling guidelines from the French Agriculture and Forestry Ministry (decree 87849) and EC Directive 86/609/EEC for animal experiments.

Rats were randomly assigned in two groups: epileptic (SE, n = 20) and control (CTRL, n = 10). One day prior to SE induction, all animals received a subcutaneous lithium injection at 128 mg/kg (lithium chloride, Sigma-Aldrich) after a light anesthesia with isoflurane. The day after (D0), all rats were given first an intraperitoneal (ip.) injection of atropine (atropine sulfate salt, Sigma-Aldrich) at 10 mg/kg. Fifteen minutes later, rats received a second ip. injection: pilocarpine (pilocarpine hydrochloride, Sigma-Aldrich) at 40 mg/kg for the EG ones and NaCl for the CT ones. NaCl volume was calculated to be equal to the mean pilocarpine injection volume. If the first pilocarpine dose was not sufficient to induce a SE, i.e. seizure that lasted more than 2 min, half doses were given every 20 min until the induction. SE was stopped after 2 h by injection of diazepam at 5 mg/kg and phenytoin at 15 mg/kg. CT rats also received ip. injection of diazepam and phenytoin, 2 h after NaCl injection.

### Tissue sample preparation

Tissues samples were obtained from SE and CTRL rats at each time points: 5 (D5), 10 (D10), 20 (D20) and 40 days (D40) after pilocarpine or saline injection (for two rats by group and condition, i.e. 16 in total). Animals that received more than 80 mg/kg of pilocarpine were excluded from the study. We included one rat that received exactly 80 mg/kg pilocarpine to achieve balanced biological replicates (n = 2) at the D10 time point, matching the other time points. No other selection criteria were applied beyond the pilocarpine dose threshold and survival. All rats that reached the D40 time point exhibited spontaneous seizures, confirming successful epileptogenesis. The experimental design with age-matched controls at each time point ensures that epileptogenesis-related changes are distinguishable from purely age-dependent effects in cross-sectional comparisons. Rats were anesthetized with Euthasol® (0.5–0.8 mL, 1/10^e^ dilution in NaCl, TVM) before decapitation. The brain was obtained through dissection and separated into two hemispheres. Immediately after, hemispheres were frozen in isopentane cooled on dry ice and stored at − 80 °C. The right hemisphere was embedded in Optimal Cutting Temperature medium and cryo-sectioned coronally on a cryostat (Leica CM 3050 S). Precisely, the hemisphere was cut until the hippocampus was complete at an anteroposterior level between − 3.14 and − 3.3 mm from Bregma (according to Paxinos and Watson’s Rat Brain Atlas), then it was trimmed with a razor blade until a section fitted into a 6 mm wide square, with the hippocampus on its center. This level was chosen because it captures key hippocampal subregions (CA1, CA2, CA3, dentate gyrus) along with adjacent cortical areas within a single Visium capture area, enabling comprehensive spatial analysis of relevant brain structures affected in temporal lobe epilepsy. A 10 µm tissue section was layered on one capture area of a Visium Gene Expression Slide following the operational guidelines of the Visium platform (10× Genomics). The Visium Gene Expression slide comprised four capture areas, each constituted of a 6.5 mm wide square with approximately 5000 barcoded spots (55 µm wide with each center separated by 100 µm). Tissues sections were distributed carefully to avoid the batch effect. Visium slides with the sections were fixed, stained, and imaged with Hematoxylin and Eosin (supplementary Fig. 1) using a 20× objective on an NanoZoomer S60 (Hamamatsu, Japan). Tissue was then permeabilized for 18 min, which was established as an optimal permeabilization time based on tissue optimization time-course experiments. The poly-A mRNAs from the slices were released and captured by the poly(dT) primers and precoated on the slide, including a spatial barcode and a Unique Molecular Identifiers (UMIs). After reverse transcription and second-strand synthesis, the amplified cDNA samples from the Visium slides were transferred, purified, and quantified for library preparation. The total RNA yield obtained were between 176.55 and 868.30 ng for 40 µL (Agilent Bioanalyzer High Sensitivity DNA kit).

### Spatial gene expression library preparation and sequencing

Sequencing libraries were prepared according to Visium Spatial Gene Expression Reagent Kits User Guide (CG000239 Rev D, 10X Genomics, 2020). Libraries were sequenced on an Illumina NovaSeq 6000 sequencer. All samples (n = 16) were equimolarly pooled and loaded at 1.5 nM final on an Illumina S2-100 cycles cartridge (4100 million reads). Sequencing was done by the pair-end method and with the following parameters: 28 cycles for Read 1, 88 cycles for Read 2 and 10 cycles for each index. Visium data, raw FASTQ files and images were processed with Space Ranger software v1.2.2 to produce spot by genes count matrix and position of spots on tissue image.

### Visium data processing and expression quantification

Demultiplexed read 2 FASTQ files were trimmed to remove TSO adapters and polyA homopolymers using cutadapt 3.4 [50]. The TSO sequence (AAGCAGTGGTATCAACGCAGAGTACATGGG) and a polyA homopolymers of length 10 were trimmed as regular adapters at the 5′ end and the 3’ end of reads 2 respectively with a minimum overlap of 5 bases and a maximum number of sequences to trim set to 2. The first base at the 3′ end of reads 1 was hard trimmed. Reads 2 shorter than 20 bases after trimming were filtered out. Other parameters were set to default values. Trimmed and filtered paired FASTQ files and corresponding Hematoxylin and Eosin images were processed with Space Ranger v1.2.2 count subcommand to generate count matrices. The *Rattus norvegicus* mRatBN7.2 genome assembly [32] and RefSeq release 108 annotations have been shown to exhibit a higher degree of completeness in comparison with other *Rattus norvegicus* reference genomes when constructing a reference genome particularly in the context of hippocampal subregions [58]. Here, reads 2 were aligned to a reference genome built on these assembly and annotations using Space Ranger v1.2.2 *mkref* subcommand.. The number of Unique Molecular Identifiers (UMI) were counted. H5 matrices storing raw read counts were loaded using *Read10X_h5* Seurat v4.3 R package function [27]. A *SpatialExperiment* was created per sample [60]. Mean UMI counts and number of spots with non-null UMI counts were computed for all expressed genes. Mitochondrial genes were identified in RefSeq release 108 *Rattus norvegicus* genome with the prefix “mt-” in their gene name. Spots with less than 1000 UMI counts or less than 500 genes with non-null UMI counts were filtered out.

### Multi-slice integration and transcriptome-based spatial clustering and subclustering

For each sample, UMI counts were normalized across spots by variance stabilizing transformation using SCTransform v2 regressing out percentage of mitochondrial UMI counts [25]. To perform dimensionality reduction, multi-slice integration and clustering of spots we tested the Seurat anchor-based canonical correlation analysis (CCA) integration procedure and Harmony with several combinations of parameters (number of highly variable genes ranging from 250 to 2000, considering the first 10, 15 or 20 dimensions, and clustering resolution parameter value ranging from 0.01 to 0.5). The Seurat workflow was applied to integrated objects using the *FindNeighbors* and *FindClusters* functions. Thus, we evaluated the outcome clustering of all combinations of parameters calculating the average silhouette scores of all spots. The silhouette score measures how close a spot is to other spots of its cluster and how distant it is to all the spots of its neighboring cluster, i.e. the closest cluster [61]. The silhouette score ranges from − 1 to 1. A value close to 1 indicates that the spot was appropriately clustered while a value close to − 1 denotes that the spot would be more appropriately assigned to the neighboring cluster. The Seurat CCA integration procedure using 2000 HVGs, 10 dimensions and a resolution of 0.03 which achieved the highest silhouette score among all combinations tested and was retained. The silhouette score was also computed using the condition, time point and sample of origin to evaluate their dispersion among clusters as a silhouette score value close to 0 denotes that a spot could be equivalently assigned to neighboring clusters.

A second round of clustering was applied to the spots of each cluster independently. To do so, the procedure detailed in the previous section was applied a second time from normalization to clustering, using the UMI counts after filtering the expression matrices of all samples to the set of spots of each cluster independently. For the cortex cluster, we retained the sub-clustering obtained with 250 HVGs, 10 dimensions and a resolution of 0.1, and for the hippocampus cluster the one with 2000 HVGs, 10 dimensions and a resolution of 0.05.

### Marker identification

For the identification of marker genes for each cluster and subcluster we identified differentially expressed genes (DEG) in each cluster/sub-cluster compared to all other clusters or sub-clusters using only the CTRL samples. To do so, we applied Seurat *FindAllMarkers* function using default parameters. We fit a two-group negative binomial generalized linear model for each gene (‘test.use’ parameter set to ‘neg.binom’) regressing out the sample of origin of each spot (‘latent.vars’ parameter set to ‘orig.ident’) to take into account the different replicates which composed our dataset. We did not filter the genes to test based on their log fold-change (‘logfc.threshold’ set to 0). Considering that the counts were not log-transformed we set the function for gene average expression calculation to log2(mean(x) + 1) where x is a vector of UMI counts. The obtained *p* value was corrected by the Benjamini–Hochberg procedure to control the false discovery rate due to multiple testing.

For the Heatmap of cluster marker (Fig. 2C), we used the previously generated DEG which we filtered for a log fold change > 1. On those, the top 5 marker genes of each cluster/subcluster based on adjusted *p* values were selected for illustration using the Seurat *DoHeatMap* function and SCT assays with the following parameters dispersion between − 0.5 and 3.

### SE-associated differential expression and pathway analyses

We applied Seurat *FindMarkers* function to identify DEG between SE and CTRL samples at each time point using the same parameters as those of the *FindAllMarkers* function when used to identify cluster marker genes. In particular, we used the sample from each spot as an explanatory variable of a two-group negative binomial generalized linear model to take advantage of the two replicates per condition and time which composed our dataset. For the Heatmap DEG in pathway (Fig. 3), DEG with a *p* values < 0.05, a log fold change superior to 0 and inferior of − 1 and which is included in *Long term memory*, *Synapse pruning*, *Microglial cell activation*, *Regulation of chaperone-mediated autophagy* and *Antigen processing and presentation of exogenous peptide antigen* were selected, then the SCT matrix including those genes were z-score scaled using R scales package, the matrix was then capped between − 2.5 to 2.5 using Seurat *MinMax* function, HeatMap was produced using ComplexHeatmap R package.

SE associated pathway activities were evaluated at the level of clusters and subclusters using the SCPA v1.5.2 package [10] with Gene Ontology: Biological Process, KEGG and Hallmark genesets from the Molecular Signatures Database (msigdb package using *Rattus norvegicus* as species parameters [42]. Seurat objects were splitted between SE and CTRL conditions and extracted for each combination of cluster, subcluster, and time point using the *seurat_extract* function of the SCPA R package. The SCPA *compare_pathways* function was applied with default parameters.

### Spot-level visualization of pathway activity and cell-type annotations

Pathway activities were analyzed for each spot using the AUCell R package [1]. For each geneset, scores computed with *AUCell_buildRankings* and *AUCell_calcAUC* functions were plotted on transcriptome spots on Visium images using the *SpatialFeaturePlot* of the Seurat R package with parameter keep.scale set to “all” (slices selected are indicated in Supplementary Table 1). Cell-type annotations were computed using CARD [46]. The CARD object was created using a raw RNA count matrix and Adolescent mouse brain atlas from the Linnarson lab [84] was used as reference using SubClass annotation levels as ct.varname and SampleID as sample.varname and raw count matrix as sc_count, the minimum count per spot was set to 5 and the minimum count per gene was set to 100. Deconvolution was performed using the CARD deconvolution function with default parameters. Proportions for 134 different cell-type sub-classes were estimated for each spot. To facilitate the visualization of cell-types similar subclasses with low proportions were regrouped together into their superior subclass in the cell-type ontology graph. We retained a total of 20 different cell-types (See Supplementary Table 5). Cumulative cell-types proportions by condition and time point were then compared between SE and CTRL using R *prop.test* function with default parameters. Spot-level cell-type proportions were plotted on Visium images using the *SpatialFeaturePlot* function using keep.scale “all” for Fig. 6 and no scaling for Supplementary Fig. 8.

## Results

**Fig. 1:**
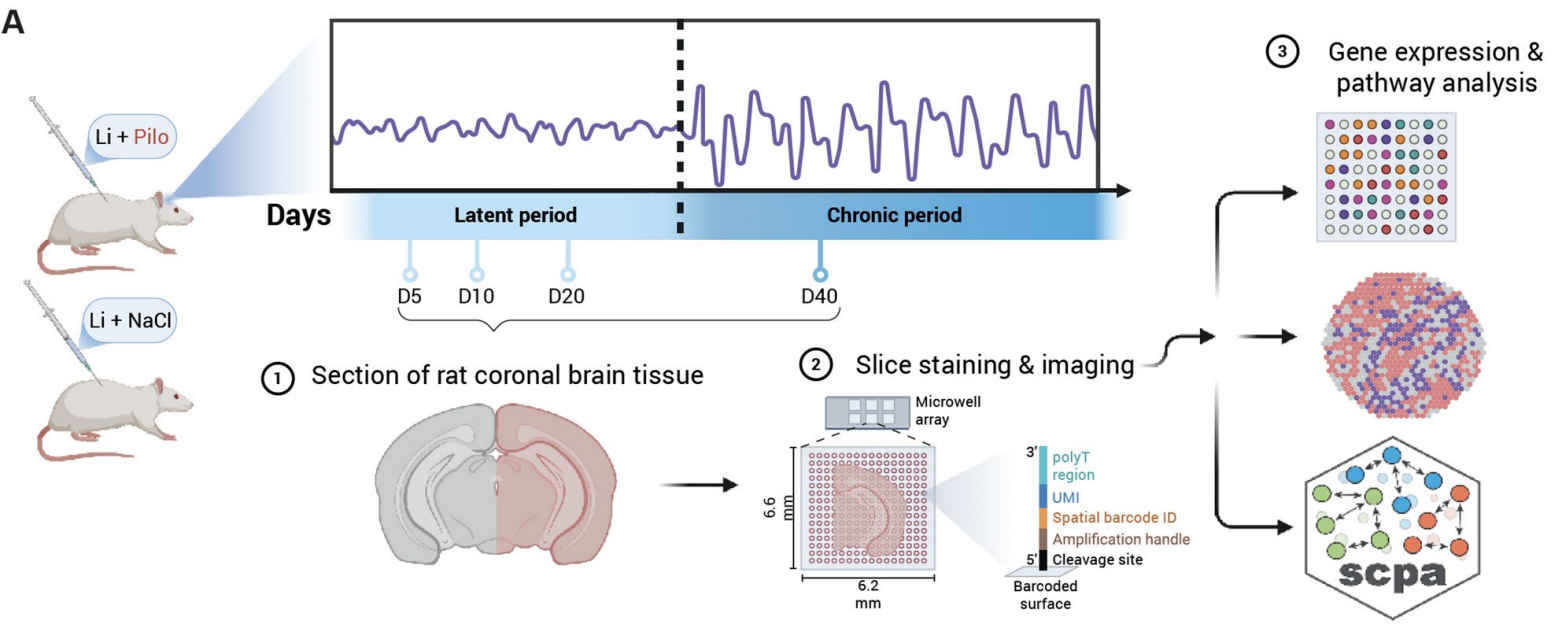
Graphical abstract of experimental method. Brains from both rodent lithium-pilocarpine-induced SE rats and lithium-control counterparts were collected at four time points to apply the Visium spatial transcriptomics technology. Clustering of spots, differential expression, pathway activity and cell-type annotations were analyzed and visualized on brain sections. Blue dots: tissue sampling time point, *Li* lithium, *Pilo* pilocarpine, *SCPA* Single cell pathway analysis; figure produced using BioRender

The overall goal of the present study was to investigate the temporal and spatial transcriptional dysregulations during epileptogenesis by applying spatial transcriptome analyses on the lithium-pilocarpine rat model of TLE at four time points of the latent and the chronic phases to characterise brain region-specific SE-associated gene expression alterations and pathway changes (Fig. 1). For this purpose, 16 frozen coronal sections of the right brain hemispheres centred on the hippocampus were selected (8 from rats that developed SE and 8 controls, 2 for each time point and condition). We performed Visium-based spatial transcriptomic sequencing and detected from 2647 to 3548 spots per sample, with each spot containing 0–10 cells. An average of 15,000 UMI were counted per spot. UMI distribution was homogeneously repeated on Visium slides except on the pyramidal layers, which have higher RNA expression levels (Supplementary Fig. 1).

### Transcriptome-based clustering and subclustering of spots retrieved anatomical brain regions

After careful multi-slice integration and dimensional reduction, all samples spots were clustered based on expression profiling of the 2000 most HVGs (Supplementary Fig. 2A, methods). When projected to tissue sections, the clusters identified well-defined spatial areas corresponding to anatomical brain regions (Supplementary Fig. 2). We annotated these areas using “The Paxinos and Watson atlas of rat brain” [55]. Cluster 0 was superimposed on the cortex. Cluster 1 encompassed the corpus callosum, internal and external capsules, the habenula, and the choroid plexus around the ventricle. Cluster 2 overlapped various thalamic nuclei. Cluster 3 corresponded to the hippocampus. Finally, cluster 4 covered the caudate putamen.

**Fig. 2:**
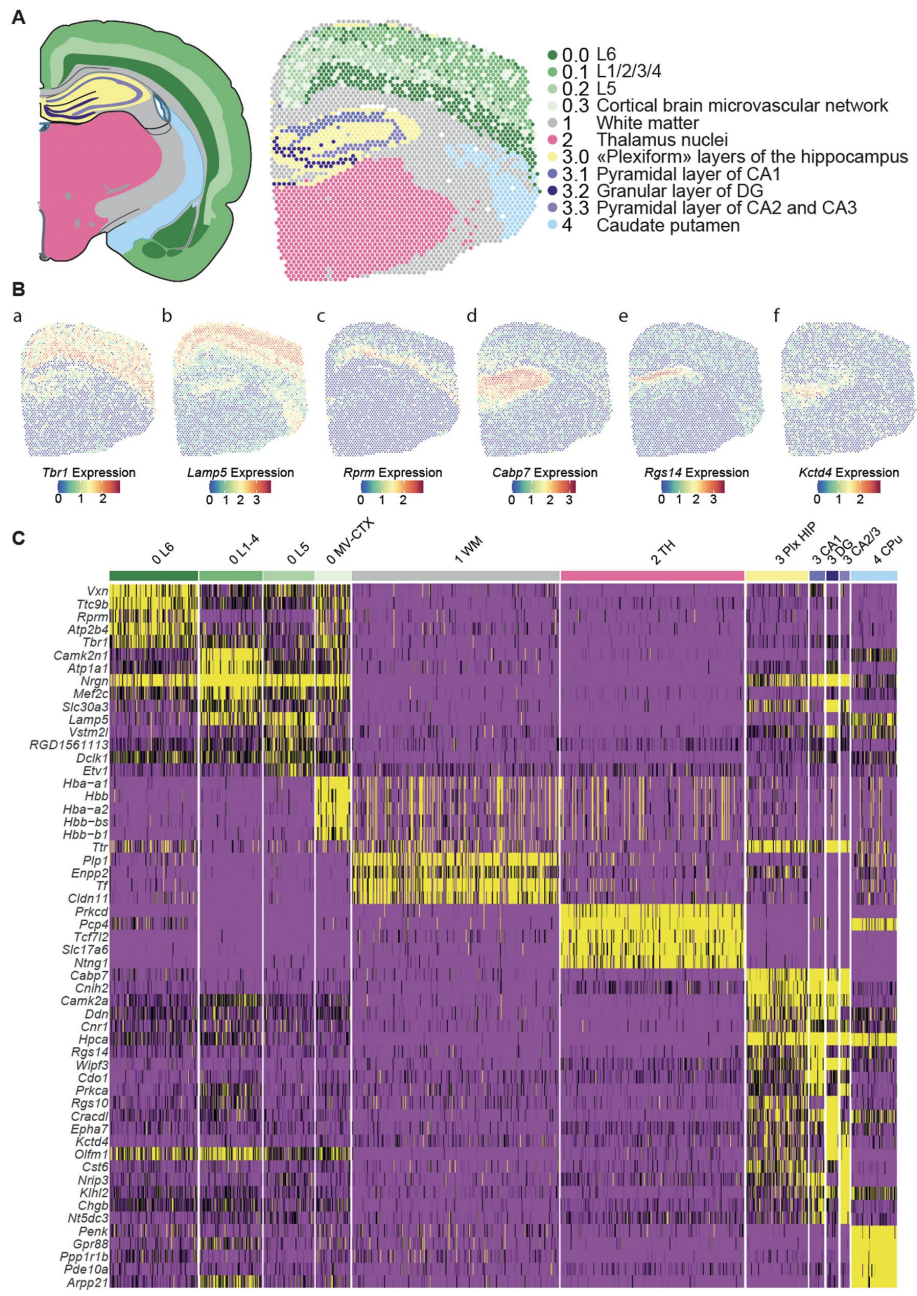
Clustering analysis. **A** Spatial location of the clusters and subclusters. On the left, visualisation of cluster localisation using Swanson, L.W. (2004) *Brain maps: structure of the rat brain*, 3rd edition slice level 28. On the right visualisation of the clusters on a Visium slide, each dot represents a spot in the sample B_L1_S2, colored by the assigned cluster. **B** Spatial expression of a selection of marker genes specific to cluster or subclusters. **a** *Tbr1* for the cortex **b** *Fezf2* for L5 cluster **c** Rprm for L6 cluster **d** *Cabp7* for “plexiform” layer **e** *Rgs14* for pyramidal layer of CA1 **f** *Kctd4* for the granular layer. Gradient represents the normalised expression of each gene on B_L1_S2 samples **C** Heatmap showing the top five enriched markers in each of the unsupervised clusters identified in the Visium sections

To obtain more detailed sub-structures on the cortex and hippocampus areas, we performed sub-clustering for previously identified clusters 0 and 3 (Supplementary Figs. 3 and 4). The clusters and sub-clusters were projected onto tissue sections at D5 and the top marker genes for each cluster/sub-cluster are shown in Fig. 2. We annotated them based on their visual overlapping with anatomical regions of “The Paxinos and Watson atlas of rat brain” and comparing their identified marker genes with “The Human Protein Atlas” database [66] and “The Allen Mouse Brain Atlas” [38] (Supplementary Tables 2 and 3).

Sub-cluster 0.0 covered both cortical cell layers 6a and b, the sub-cluster 0.1 encompassed cortical cell layers 1–4, and sub-cluster 0.2 superimposed cortical cell layer 5 (Supplementary Fig. 3). The sub-cluster 0.3 spots were scattered across the cortical areas and were annotated as a cortical microvascular network because their top marker genes corresponded to genes coding for haemoglobin chains. The sub-clustering of cluster 3 identified four spatially and histologically significant sub-clusters 3.0, 3.1, 3.2 and 3.3 corresponding respectively to the plexiform layers of the hippocampus, the CA1 pyramidal cell layer, the DG granular layer, and the CA2 and 3 pyramidal cell layers (Supplementary Fig. 4). Some of the identified marker genes are well-recognized markers for the corresponding anatomical region, such as *Trb1* for cortical layers with higher expression in layer 6 [22] (Fig. 2B and Supplementary Fig. 2D) and *Etv1* for cortical layer 5 [83]. For other identified marker genes the expression pattern previously reported in mice is concordant: *Ntng1* expression pattern was reported in mice with the highest values in thalamus [81], *Camk2a* expression was studied in mice with the most abundant expression in DG, followed by CA1 and CA3 [74], *Gpr88* was reported highly and almost exclusively expressed in caudate putamen and nucleus accumbens [20]. Another line of evidence was brought replicating the differential expression of half of the top marker genes (25/54) on coronal sections of in situ hybridisation experiments of the Allen Brain Adult Mouse Anatomical Atlas (Supplementary Table 3). Altogether, our transcriptome-based clustering and subclustering of spots successfully retrieved anatomical brain regions and known marker genes, thereby validating the newly generated spatial transcriptomic dataset and providing a solid basis for further biologically relevant investigations.

### Profound transcriptomic dysregulations associated to SE

**Fig. 3:**
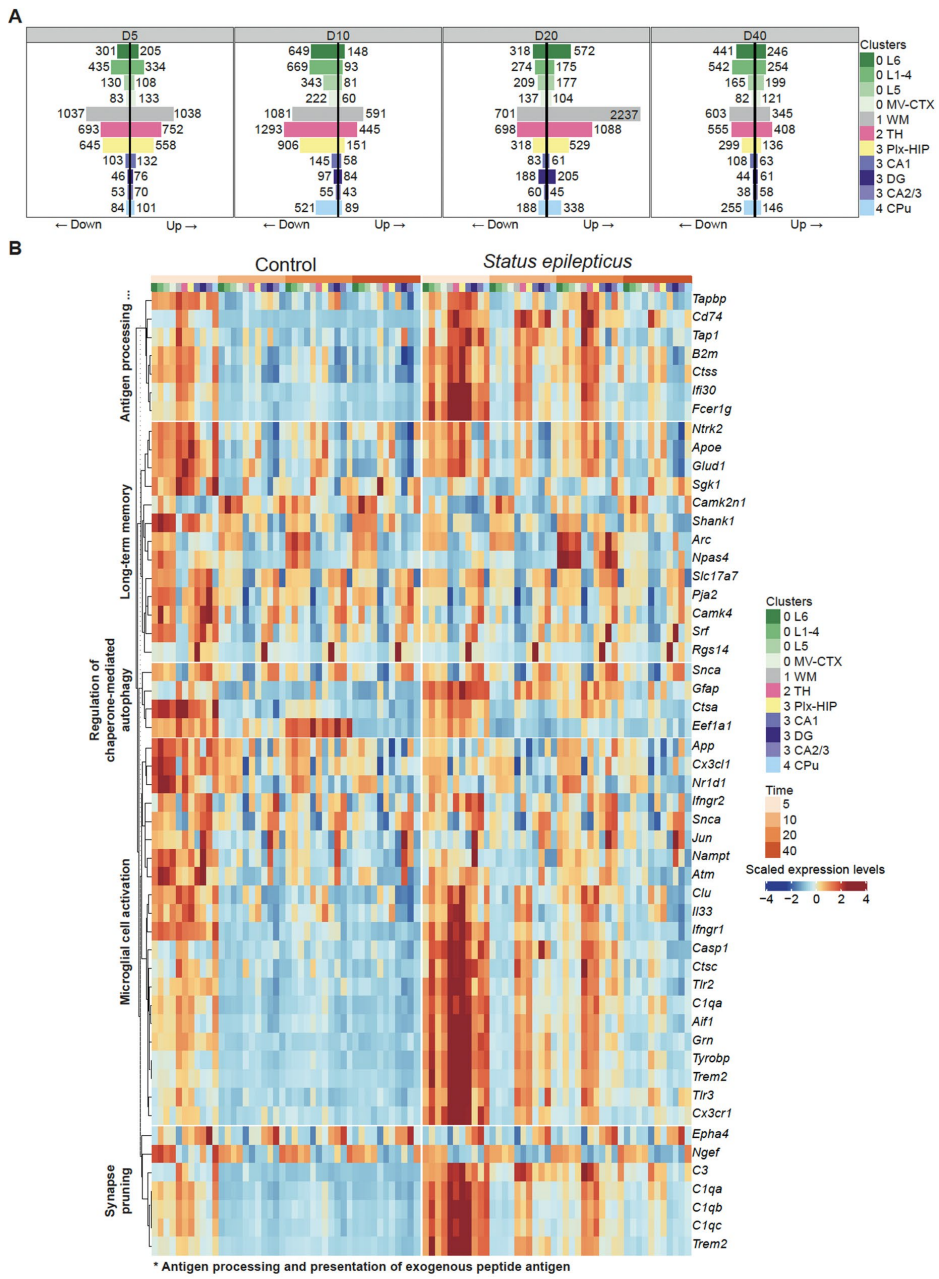
Differential expression analyses **A** Barplot of the number of SE dysregulated genes. The SE dysregulated genes correspond to genes identified as differentially expressed between SE and CTRL conditions (adjusted *p* value < 0.05). Barplots have been separated into a panel for each time point (D5, D10, D20, D40), each panel is divided into a left side, which corresponds to the downregulated in the SE condition and a right side for the upregulated ones; each bar represents a different cluster. **B** Visualization of the expression of SE dysregulated genes grouped by their Gene-Ontology (GO) annotations. Heatmap showing the average expression levels of SE dysregulated genes associated with the following GO biological process (BP) terms: *Antigen processing and presentation of exogenous peptide antigen*, *Long term memory*, *Regulation of chaperone-mediated autophagy*, *Microglial cell activation* and *Synapse pruning.* Expression values are grouped by condition, time point and cluster membership (another version of this heatmap with the same data but grouped by cluster membership is available in Supplementary Fig. 6)

Following SE, brain tissues exhibited profound transcriptional dysregulation at the four time points and in all clusters and subclusters (Fig. 3A, Supplementary Table 4, Supplementary Fig. 5).

**Fig. 4:**
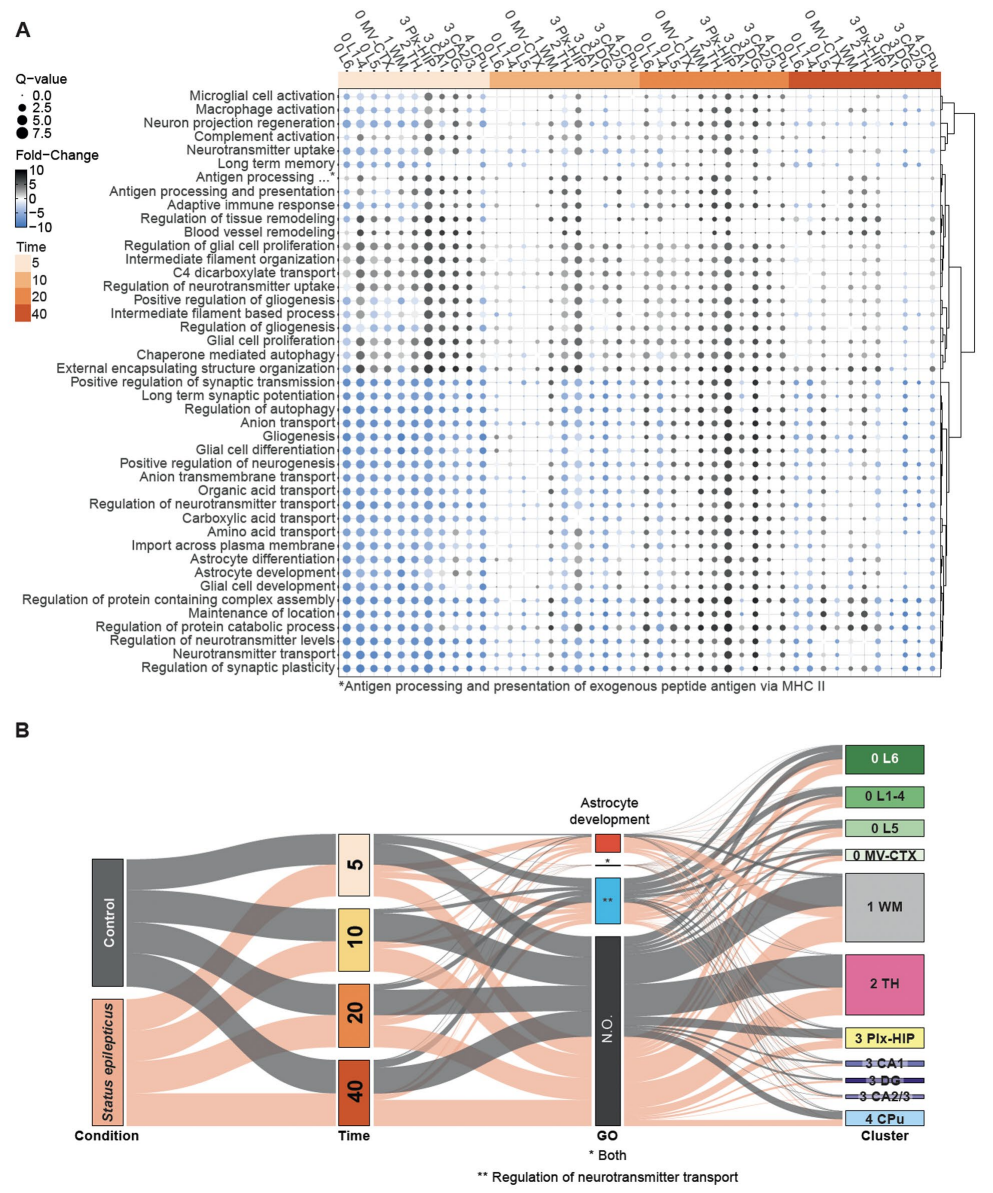
Pathway analysis **A** Dot plot of selected SCPA analysis between SE and control. **A** comparison was performed at each time point for each cluster; the blue–white–grey double colour gradient represents the direction and the magnitude of the fold-change between SE and CTRL and the dot size represents the Q-value. **B** Alluvial diagram of all spots showing their classification in several dimensions of categories: condition, time point, enrichment for selected GO:BP terms and cluster membership. Each flow represents an individual spot, colored according to its condition (grey for control and salmon color for SE). The thickness of each link between two categories represents the proportion of spots belonging to these two categories. The time categories are represented using a yellow-orange gradient from D5 to D40. The categories of enrichment selected to be represented are: *Astrocyte development (red), Regulation of neurotransmitter transport (blue)*, both terms (“Both”) and none (“N.O.”: neither *Astrocyte development* or *Regulation of neurotransmitter transport*). Spot enrichments for these terms were estimated using AUCell values calculated for those terms with a threshold of 1.8

The expression level was perturbated for a substantial number of genes: 4002 genes were significatively upregulated in SE rats in at least one of the 44 comparisons (SE vs. CTRL comparison for 11 clusters, subclusters and 4 time points) and reduced for 4154 genes (adjusted *p* value < 0.05). The same gene was consistently found upregulated in SE rats in more than two comparisons for 1430 genes, and downregulated for 1852 genes respectively. Specifically, the main genes with consistently increased expression included *Gfap*, *Tyrobp*, *Cd74*, *Aqp4*, and *Spp1*, while those with decreased expression included *Ppia*, *Ttr*, *Ubb*, *Eef1a1* and *Ndufs5*. Gene Ontology (GO) annotations of DEGs with substantial fold changes were enriched for *Microglial cell activation*, *Long term memory*, *Regulation of Chaperone Autophagy, Antigen Processing and Presentation of Exogenous Peptide Antigen,* and *Synapse pruning* (Fig. 3B and Supplementary Fig. 6). Transcriptomic dysregulations associated to SE were mainly observed at the later part of the latent phase (D20) and more strongly in cluster 1 (white matter), and cluster 2 (thalamus) and subcluster 3.2 (Figs. 3A and 4A).

In the sub-cluster 3.2 (DG) the expression of the transcription factor *Srf* and immediate early genes *Npas4*, *Arc*, and *Jun* were highly increased (Fig. 3B). To identify SE perturbed pathways we used the SCPA R package. This approach relies on a graph-based, nonparametric statistical framework that compares the multivariate distributions of gene sets between different conditions. Applying SCPA for each cluster and subcluster across the four time points revealed strong pathway dysregulations in SE samples (Fig. 4). The highest SE-associated activity changes were observed at D5 across all investigated regions, and in hippocampal sub-clusters 3.0 and 3.2 at D20. Pathways related to *Long term synaptic potentiation, Neurotransmitter transport* and *Regulation* of *synaptic plasticity* showed significantly lower activity in SE than in CTRL samples in all clusters and subclusters at D5. The same pathways exhibited higher activity in the white matter cluster 1 at D10, spreading to some cortical and hippocampal sub-clusters (0.1, 0.3, 3.0, 3.2) and to caudate putamen (cluster 4) at D20. Pathways related to *Blood vessel remodeling*, *Glial cell proliferation*, *Chaperone mediated autophagy* and *External encapsulating structure organization* were more active in SE in almost all clusters and subclusters (0.1, 0.2, 0.3, 1, 2, 3.0, 3.1, 3.2, 3.3) at D5, mainly in thalamus (cluster 2) and in the plexiform layers of the hippocampus (sub-cluster 3.0) at D10, plus in granular layer of DG (subcluster 3.2) at D20. Pathways related to *Astrocyte development, Microglial cell activation* and *Adaptative immune response* displayed a pattern of dysregulations with lower activity in SE in cortical clusters, white matter and thalamus (subcluster 0.0, 0.1, 0.2, 0.3, cluster 1 and 2) and increased activity in hippocampal subclusters at D5, followed by a higher activity in DG and the plexiform layers of the hippocampus (subclusters 3.0 and 3.2) at D20.

### Spatial visualization of SE associated transcriptomic dysregulations revealed localized microglial activation and reactive astrogliosis

**Fig. 5:**
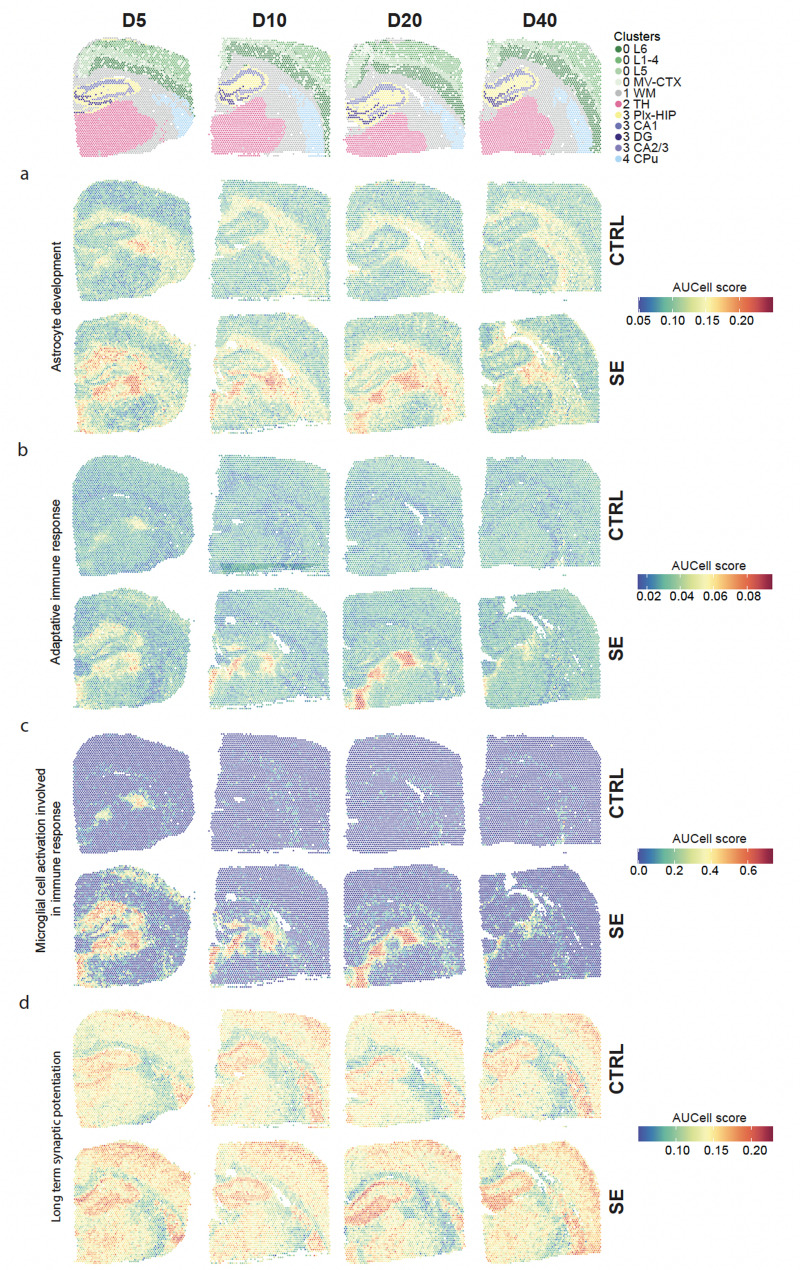
Spatial pathway activity. Visualisation of pathway activity calculated using AUCell on selected GO:BP terms **a** Adaptive immune response **b** Astrocyte development **c** Microglial cell activation involved in immune response **d** Long-term synaptic potentiation

We examined the expression distribution of previously identified SE-perturbed pathways across brain sections on Visium images. For this purpose, AUCell scores were computed for all spots of all samples and projected on Visium images, to evaluate the relative expression for each tested pathway across the whole experiment (see “Methods” section). Spots with AUCell scores above 1.8 for the *Astrocyte development* GO biological process were near exclusively found in SE samples and mainly at D5 in the white matter and in the thalamus (cluster 1 and 2, Figs. 4B and 5A).

Spatial visualization of several SE-perturbed pathways associated with reactive astrogliosis exhibited a similar pattern of distribution with high AUCell scores at D5, D10, D20 and lighter at D40 (*Astrocyte development, Adaptive immune response*, and *Microglia cell activation involved in immune response* GO biological processes, Fig. 5A–C). At D5, this area of reactive astrogliosis surrounded the granular layer of DG, particularly in the CA1 field, spreading out beyond the hippocampus in white matter and in parts of the thalamus and in the L1/2/3/4 cortex cluster 0.1. At D10 and D20, the signal remained high mainly in the hilus of DG and in three patches of the thalamus, similarly localized across the SE samples, suggesting this SE-associated reactive astrogliosis occurred in specific thalamic nuclei. For other pathways, the distributions of AUCell scores showed strong region-specific patterns in all conditions, with slightly SE-associated changes concurring with the previous SCPA results (Fig. 5D, Supplementary Fig. 7). For the *Regulation of neurotransmitter transport* GO biological process high AUCell scores covered neocortical and nuclear-rich hippocampal layers with a loss of signal in SE samples compared to CTRL more highly pronounced at D5 (Fig. 4B, Supplementary Fig. 7C). For the *Long term synaptic potentiation*, GO biological process high AUCell scores superimposed the granular layer of DG and in the pyramidal layer of CA1, CA2 and CA3 in both CTRL and SE samples, with higher signal in SE samples at D20 (Fig. 5D).

### Cell-type composition alterations

**Fig. 6:**
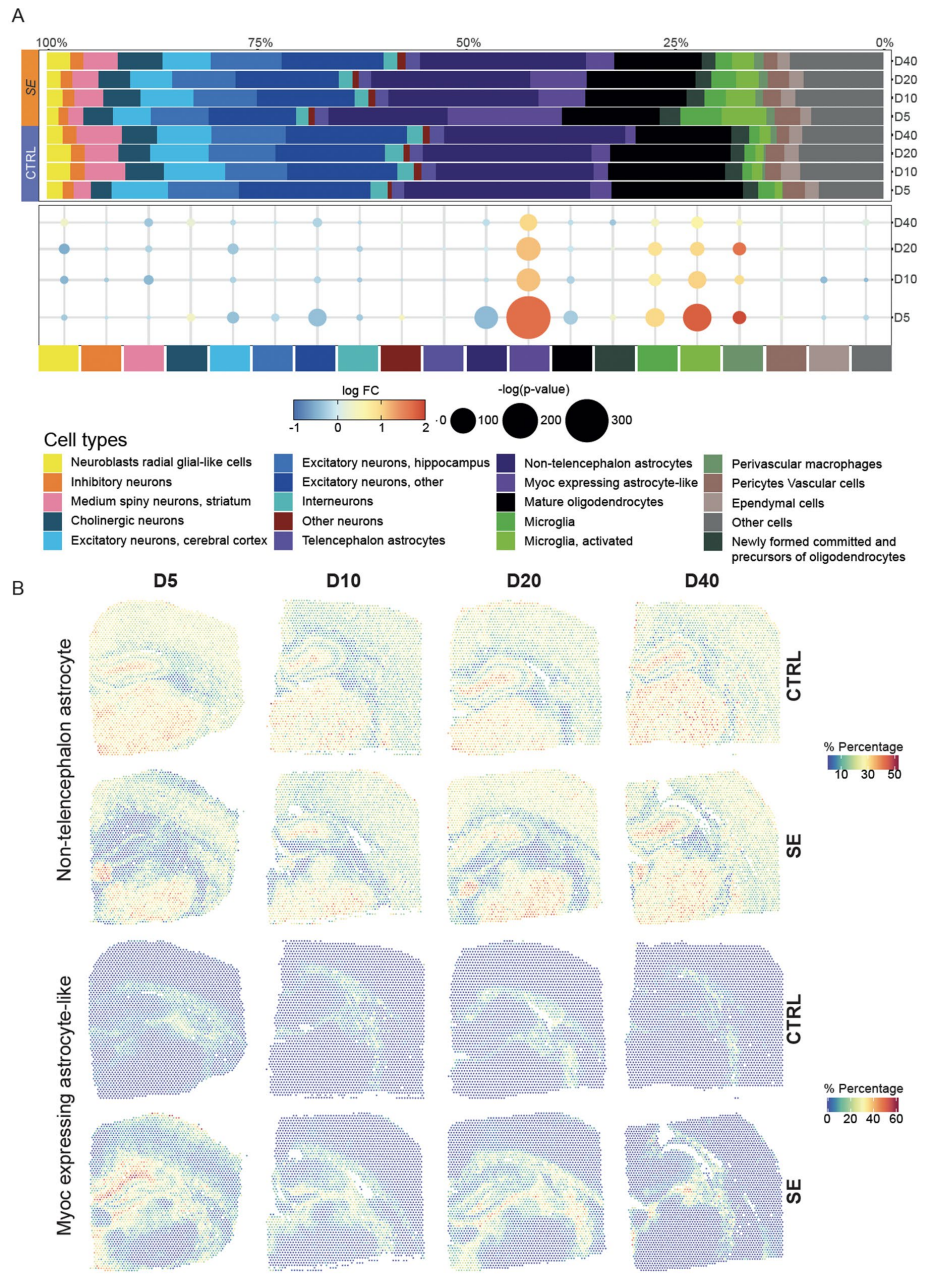
Cell-types deconvolution. **A** Cell-type composition by condition and time point. The top bar plot shows the cumulative estimated proportions of cell-types grouped by condition and time point. Each bar represents the average of two sections as biological replicates (the sample-by-sample equivalent bar plot is available in Supplementary Fig. 9). In the dot plot below, dot size represents the significance of cell-type proportion differences between SE and CTRL (z-test), while the color gradient indicates the scaled magnitude of these differences ranging from reduced (blue) to increased (red) proportions. **B** Spatial composition of selected cell types. Visualisation of the percentage of cell types per spot for selected cell types *Non-telencephalon astrocyte* and *Myoc expressing astrocyte*

To investigate the cell type composition of the rat brain undergoing epileptogenesis, we transferred available annotations of the cell-type diversity of the mouse brain [84] using CARD, a reference-based deconvolution method for spatial transcriptomics [46]. After simplification into 20 main different cell-types we compared the estimated cell-type proportions between SE and CTRL animals (Fig. 6 and Supplementary Fig. 8).

Spot-level proportions projected on Visium images of SE sections at D5 displayed cell-type specific distributions (Supplementary Fig. 9). We observed a reduction of the proportion of neurons under epileptic conditions with a decrease of *excitatory neurons, cerebral cortex* and *other* at D5 (z-test, *p* value 1.00 × 10^−7^ and 4.14 × 10^−18^ respectively). Changes in cell type composition were also observed for astrocytes, oligodendrocytes and brain-resident myeloid cells with a strong increase for *Microglia, activated* at D5 (z-test, *p* value 1.91 × 10^−45^). The most important changes were identified for astrocytes with a strong increase at all time points for *Myoc expressing astrocyte-like* (z-test, *p* value 3.15 × 10^−112^ at D5) and conversely a decrease for *Non-telencephalon astrocytes* at D5 (z-test, *p* value 1.41 × 10^−30^). Spot-level proportions projected on Visium images for these two last cell-type annotations exhibited complementary distributions in each sample. The comparison between SE and CTRL sections retrieved the global reversed increase and decrease of proportions previously observed for these cell-types (Fig. 6B). In SE sections, while percentages of *Non-telencephalon astrocyte* were reduced in nearly all regions, a small, delimited area that seems to correspond to the lateral habenula exhibited high percentages. Spatial visualization of *Myoc expressing astrocyte-like* proportions on SE sections retrieved at similar pattern of distribution described for reactive astrogliosis pathways. This area included the hippocampus, the white matter cluster and thalamus parts. Together, these results suggest that after the immediate acute phase, SE led to cell recruitments, proliferation and/or differentiation in localized areas of hippocampus, white matter and thalamus that could play a role in epileptogenesis and trigger chronic seizures.

## Discussion

This study employed Visium-based spatial transcriptomics coupled with multiple analytical approaches to investigate the pathophysiological mechanisms underlying epileptogenesis comprehensively. Our findings successfully identified anatomically relevant clusters and subclusters correspondeding to known brain regions and established marker genes in control animals. Comparative transcriptome profiling revealed profound molecular alterations associated with status epilepticus (SE) across latent and chronic phases in all examined brain regions. Importantly, finer spot-level spatial visualization of SE-associated pathways and cell-type annotations revealed reactive astrogliosis extending beyond traditional hippocampal regions to encompass white matter tracts and thalamic nuclei.

While previous transcriptomic studies of epileptogenesis have predominantly focused on hippocampal subfields, often examining single time points [11, 18, 26, 43, 48, 73] recent advances have begun to reveal the broader anatomical scope of epileptogenic processes. A comprehensive single-cell and single-nucleus sequencing study examining hippocampus, temporal cortex, and thalamus across all three phases of epileptogenesis demonstrated significant thalamic gene expression changes during the latent phase [76]. Our spatial transcriptomic approach extends these findings by providing detailed visualization of reactive astrogliosis across multiple time points, revealing specific localized activation patterns in hippocampus, white matter, and distinct thalamic regions that could correspond to the mediodorsal, central lateral, and laterodorsal nuclei. This tissue reorganization persisted throughout the latent phase but disappeared during the chronic phase. While astrogliosis and the active role of astrocytes in epilepsy pathophysiology have been extensively characterized in hippocampal regions, evidence for similar mechanisms in thalamic nuclei remains limited [72]. Previous studies have documented thalamic structural and functional abnormalities with GFAP-reactive astrocytes in the ventrobasal nucleus following neocortical focal status epilepticus [49], and the reticular nucleus has been implicated in absence seizure mechanisms [14]. Current evidence increasingly positions the thalamus as a dynamic regulator within epileptic networks rather than a passive relay structure. Its critical role in facilitating generalized seizure propagation and secondary generalization of focal epilepsies highlights its capacity to actively shape pathological synchronization [41, 65]. The consistent observation of thalamic atrophy in MRI studies of TLE, particularly ipsilateral to the seizure focus, suggests that thalamic involvement is not merely a consequence of cortical pathology but may evolve alongside the epileptogenic process itself [80, 82]. These findings also raise the possibility that progressive thalamic remodeling exacerbates network dysfunction and clinical severity. While the clinical efficacy of thalamic neurostimulation underscores its central role, the mechanisms by which stimulation modulates pathological activity remain only partially understood and may depend on nucleus-specific connectivity [71].

Our integration of Visium spatial transcriptomics with reference mouse brain single-cell sequencing data revealed an inverse complementary organization of two astrocytes classes (termed *Non-telencephalon astrocytes* and *Myoc expressing astrocyte-like*) with pronounced alterations in SE animals. Astrocyte heterogeneity and the characterization of distinct subtypes and functional states remain active areas of investigation [29]. The Linnarsson laboratory reference dataset, generated from micro-dissected brain tissue regions, had distinguished seven different astrocytes subclasses within and outside telencephalon. [84]. However, it is important to note that these anatomical classifications may not perfectly align with functional astrocyte states, and the molecular patterns defining *Non-telencephalic astrocytes* in this reference dataset may overlap with more widespread astrocyte functional subtypes present in hippocampal tissue. The highly specialized astrocytes of the dorsal midbrain, termed *Myoc expressing astrocyte-like* cells were recently characterized as the glia limitans superficialis (GSL) astrocytes [28]. GSL astrocytes reside on the surface of the central nervous system with processes that extend into the parenchyma. Their specific localization pattern closely corresponded to the projection of the *Myoc expressing astrocyte-like* cell-type proportions in CTRL sections (Fig. 6B). Significantly, GSL astrocytes constitutively express baseline reactive transcripts, making them prime candidates for explaining the observed expansion in SE sections. Whether this expansion results from GSL astrocytes proliferation or differentiation of neighboring parenchymal glial cells remains to be explored. In human electrode-mapped TLE surgical samples, abnormal prominent glial subpopulations were described with a dedifferenciated hybrid signature of both reactive astrocyte and OPC markers [53]. A combination of longitudinal single-cell or single-nuclei multi-omics, with higher-resolution spatial transcriptomics, consensus brain cell-type classifications, patch-sequencing, lineage tracing methods and functional validation of these molecularly identified cell subtypes will be needed to fully elucidate mechanistic cell–cell interactions and each cell lineage’s contribution during epileptogenesis.

For brain resident myeloid cell-types, we observed significant SE-associated changes in proportions and pathway activities, corroborating their active role during the latent phase of epileptogenesis. Microglia maintain brain homeostasis and support neuronal functional networks through multiple mechanisms, including structural resolution of beaded dendrites, phagocytosis, and regulation of astrocytic activation [5, 8, 21]. While the proconvulsive effect of microglial was demonstrated in the acute phase following SE in chemoconvulsant-induced epilepsy models, their sustained activation in the latent phase appears to exert more balanced effects, potentially providing neuroprotection [8, 63]. However, many of these studies have focused exclusively on the hippocampus. In contrast, our results demonstrate that microglial activation encompasses a significantly broader anatomical distribution, including the hippocampus, white matter, and multiple thalamic nuclei. The functional consequences of microglial activation likely vary depending on regional context, though whether neuroprotective effects occur uniformly across all affected regions or are region-specific remains to be determined.

Although the peak of microglial activation and reactive astrogliosis was raised just after the acute phase, other transcriptomic alterations associated with SE were preferentially observed during the later stages of the latent phase (D20). For example, in the DG, *Long term synaptic potentiation* activity, and expression of immediate early genes *Npas4*, *Arc* increased at this time point, while *Srf* exhibited sustained upregulation at both D20 and D40 (Figs. 3B, 4A and 5D). The transcription factor *Srf* plays essential roles in immediate early gene expression, memory formation, synaptic function, and epilepsy-associated mossy fiber sprouting and inflammation [3, 37, 44]. Notably, *Srf* exhibits complex, cell-type-specific functions in epileptogenesis. While *Srf* deficient astrocytes provides neuroprotection in kainic acid-induced excitotoxicity models, forebrain-specific deletion of *Srf* in glutamatergic neurons pilocarpine-induces models reduced SE occurrence. However, it paradoxically increased spontaneous recurrent seizures [44, 69]. These findings illustrate the necessity for in-depth investigations into the temporal dynamics and the region- and cell-type-specific mechanisms governing the molecular pathophysiology of epileptogenesis, to rigorously define such a dual role that could represent a key target for therapeutic development.

Several limitations must be acknowledged when interpreting our findings. First, the lithium-pilocarpine model of TLE in young adult rats, despite of its wide acceptance to investigate epileptogenesis, may not fully recapitulate the complexities of human epilepsy [15, 40]. Acute lithium or diazepam administration can alter the excitatory/inhibitory brain homeostasis and transcriptional profiles [17, 31, 34, 78], making it challenging to distinguish medication-related effects from epileptogenesis-specific changes. Future studies should therefore test other ages and include appropriate controls without lithium or diazepam treatment to better disentangle age-related and drug-induced molecular alterations from those intrinsic to the epileptogenic process itself. Additionally, the newly generated spatial transcriptome dataset was analyzed using methods developed initially for single-cell RNA-seq which do not fully exploit spatial information. While our strategy of defining spatial clusters and investigating transcriptional changes through these clusters provided valuable global insights, identifying of localized transcriptional changes required spot-level analyses and projection onto Visium images. Emerging methods designed explicitly for spatial transcriptomics could enhance the detection of such localized changes [33, 36]. Our cluster generation approach prioritized objective parameter optimization based on overall mean silhouette scores. However, recent benchmarking studies have highlighted limitations of silhouette scoring for assessing data integration in single-cell studies and proposed alternative evaluation metrics [59]. Despite these methodological considerations, converging our clusters and subclusters with established anatomical brain regions and concordant marker gene expression validates our analytical approach.

In conclusion, our study provides key insights into epileptogenesis’s cellular and molecular spatio-temporal complexity, revealing a significant expansion of the anatomical regions affected by microglial activation and reactive astrogliosis during the latent phase. While these glial alterations were previously well-characterized in the hippocampus, our findings demonstrate their extension into white matter tracts and thalamic nuclei, highlighting the widespread nature of epileptogenic processes beyond traditionally recognized limbic structures. Future investigations are warranted to elucidate the relationships between these glial perturbations and neuronal homeostasis within functional networks. Although synaptogenesis and mossy fiber sprouting in the dentate gyrus have been extensively studied, the nature and functional significance of neuronal alterations in thalamic nuclei remain largely unexplored. The dual role of cellular and genetic factors in the latent phase adds considerable complexity to our understanding, making distinguishing neuroprotective mechanisms from epileptogenic processes challenging. This complexity underscores the need for more nuanced approaches to identify specific therapeutic targets for both treatment and prevention strategies in temporal lobe epilepsy.

## Supplementary Information

Below is the link to the electronic supplementary material.

Supplementary Material 1: Supplementary Fig. 1. Histological and quality control visualisation. Hematoxylin-and-eosin-stained tissue sections and corresponding spatial distribution of UMI counts per spot for each sample.

Supplementary Material 2: Supplementary Fig. 2. Whole clustering. **A** Four panels of UMAP representation of all spots passing quality control colored according to clusters, condition (treated or control), time points or sample of origin (from top to bottom and from left to right). The mean silhouette score is indicated in the top of each plot. **B** Spatial plots of clusters on tissue slices of samples at the D5 time point (treated samples on top, control samples on bottom). **C** and **D** Example of hippocampal and cortical marker (*Cabp7* and *Tbr1*, respectively) expression levels after log2 transformation. Violin plots per cluster (top), spatial plots on the tissue slice of the control samples at the D5 time point (bottom).

Supplementary Material 3: Supplementary Fig. 3. Sub-clustering of the cortex. **A** Four panels of UMAP representation of cortical cluster spots colored according to subcluster, condition (treated or control), time points or sample of origin (from top to bottom and from left to right). The mean silhouette score is indicated in the top of each plot. **B** Spatial plots of sub-clusters on tissue slices of samples at the D5 time point (treated samples on top, control samples on bottom). **C** and **D** Example of cortical layers 6a and 5 marker (*Mef2c* and *Rprm*) expression levels after log2 transformation. Violin plots per cluster (top), spatial plots on the tissue slice of the control samples at the D5 time point (bottom).

Supplementary Material 4: Supplementary Fig. 4. Sub-clustering of the hippocampus. **A** Four panels of UMAP representation of hippocampal cluster spots colored according to subcluster, condition (treated or control), time points or sample of origin (from top to bottom and from left to right). The mean silhouette score is indicated in the top of each plot. **B** Spatial plots of sub-clusters on tissue slices of samples at the D5 time point (treated samples on top, control samples on bottom). **C** and **D** Example of CA1 and DG marker (*Rgs14* and *Kctd4*) expression levels after log2 transformation. Violin plots per cluster (top), spatial plots on the tissue slice of the control samples at the D5 time point (bottom).

Supplementary Material 5: Supplementary Fig. 5. Visualisation of conditions and samples repartition by clusters. **A** Conditions repartition by clusters. On the left, the repartition of the different clusters for each condition and time, with the number of spots for each condition on the top, and on the right, the repartition of the different conditions and times for each cluster, with the number of spots for each cluster on the top. **B** Samples repartition by clusters. On the left, the repartition of the different clusters for each sample, with the number of spots for each sample on the top, and on the right, the repartition of the different samples for each cluster, with the number of spots for each cluster on the top.

Supplementary Material 6: Supplementary Fig. 6. Heatmap of SE dysregulated genes grouped by cluster membership and GO:BP annotation. Heatmap showing the average expression levels of SE dysregulated genes associated with the following GO Biological Process terms: *Antigen processing and presentation of exogenous peptide antigen*, *Long term memory*, *Regulation of chaperone-mediated autophagy*, *Microglial cell activation* and *Synapse pruning.* Expression values are grouped by condition, cluster membership and time point. The SE dysregulated genes correspond to genes identified as differentially expressed between SE and CTRL conditions.

Supplementary Material 7: Supplementary Fig. 7. Spatial pathway activity. Visualisation of pathway activity calculated using AUCell on selected GO:BP terms **a** Neuron projection regeneration **b** Regulation of neurotransmitter uptake **c** Regulation of neurotransmitter transport **d** Intermediate filament organisation.

Supplementary Material 8: Supplementary Fig. 8. Spatial deconvolution at D5 after SE. Visualisation of the percentage of cell types per spot for the selected cell types on the Visium section A_L1_S1.

Supplementary Material 9: Supplementary Fig. 9. Cell-type composition by section. Barplots representing the cumulative estimated proportions of cell-types for all spots of each section.

Supplementary Material 10: Supplementary Table 1. Sample metadata. Table summarizing the characteristics of brain slides used in this study, including: samples, stages, conditions, slides, numbers of spots.

Supplementary Material 11: Supplementary Table 2. Marker genes of clusters and subclusters. One cluster/subcluster vs all other clusters/subclusters comparisons were made to identify marker genes.

Supplementary Material 12: Supplementary Table 3. Validation of maker genes in the Allen Brain Mouse Adult in situ hybridization Atlas using the differential search web tools (https://mouse.brain-map.org/). Among the 5 top marker genes of each cluster and sub-cluster obtained using the Seurat *FindAllMarkers* function (see methods) about an half (25/54) are also listed within the top 100 differentially expressed genes using in situ hybridization experiments reported in the Allen Brain Mouse Adult Atlas.

Supplementary Material 13: Supplementary Table 4. Differentially expressed genes between SE and control samples. Cortex and hippocampus sub-clusters and clusters 1, 2 and 4 were considered for treated vs control samples comparisons. Differentially expressed genes were identified for all the clusters and sub-clusters independently.

Supplementary Material 14: Supplementary Table 5. Simplification of 134 cell-type subclasses from the Linnarson lab Adolescent mouse brain atlas into 20 cell-types.

## Data Availability

All the data of this study are publicly available and have been deposited into public repositories. All raw sequencing data and associated metadata are available in the Gene Expression Omnibus Data portal under accession number GSE301260. Code used for this analysis is available at (https://github.com/INSERM-U1141-Neurodiderot/spatial-transcriptome-epilepsy).

## Abbreviations

CT: Control group
D*x*: *x* Day after SE induction
DG: Dentate gyrus
DEG: Differentially expressed genes
EG: Epileptic group
HVG: Highly variable genes
IPI: Initial precipitating injury
ip.: Intraperitoneal
PCA: Principal component analysis
SE: *Status epilepticus*
TLE: Temporal lobe epilepsy
UMI: Unique molecular identifier
