# Supplementary figures and images for: Spatiotemporal transcriptomic mapping reveals region-specific glial activation and astrocyte shifts in epileptogenesis beyond the hippocampus
Source: Acta Neuropathol Commun. 2026 Jan 15;14:38. doi: 10.1186/s40478-026-02224-y (PMC12892793; doi:10.1186/s40478-026-02224-y)

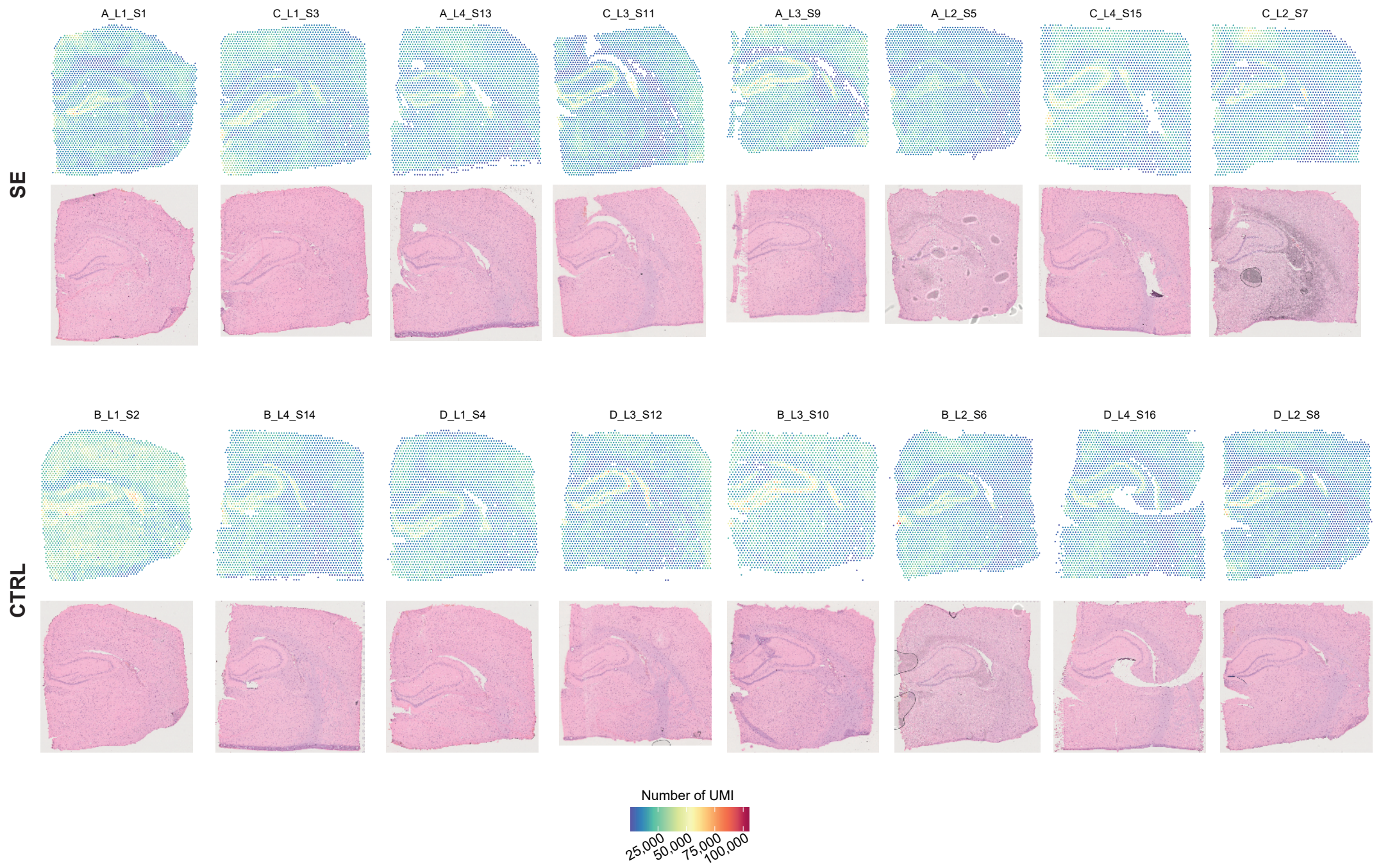

Supplementary Figure 1

Supplement: Supplementary file 1 — Supplementary Material 1: Supplementary Fig. 1. Histological and quality control visualisation. Hematoxylin-and-eosin-stained tissue sections and corresponding spatial distribution of UMI counts per spot for each sample. [file 40478_2026_2224_MOESM1_ESM.pdf]

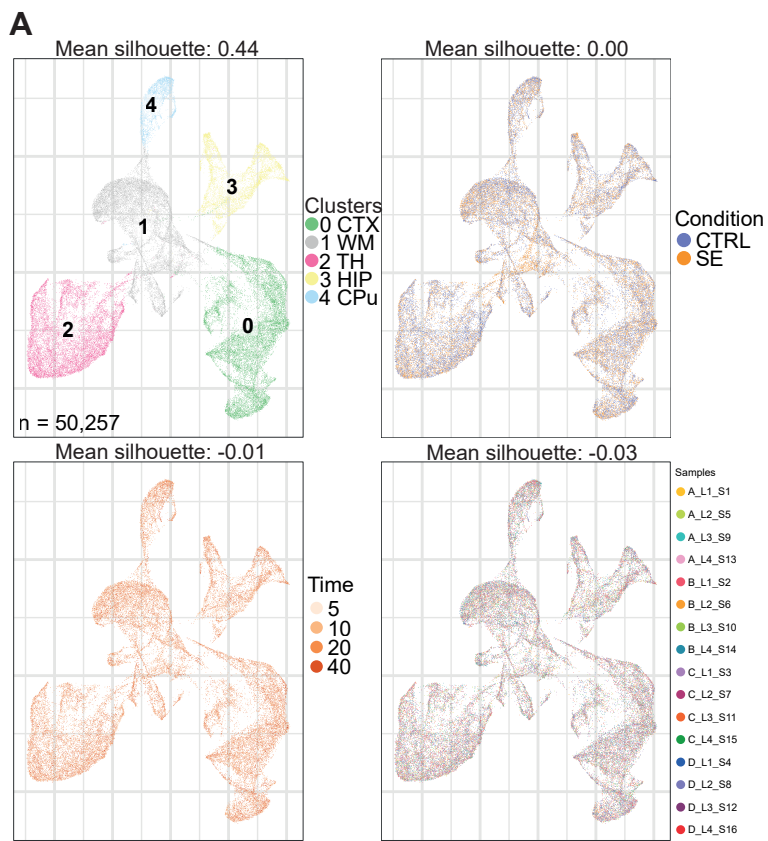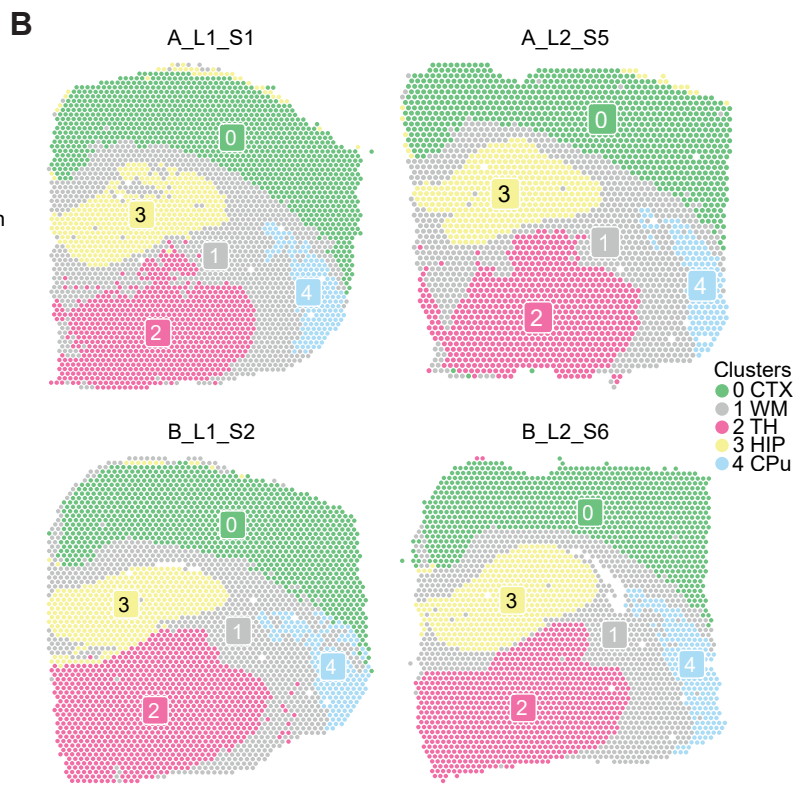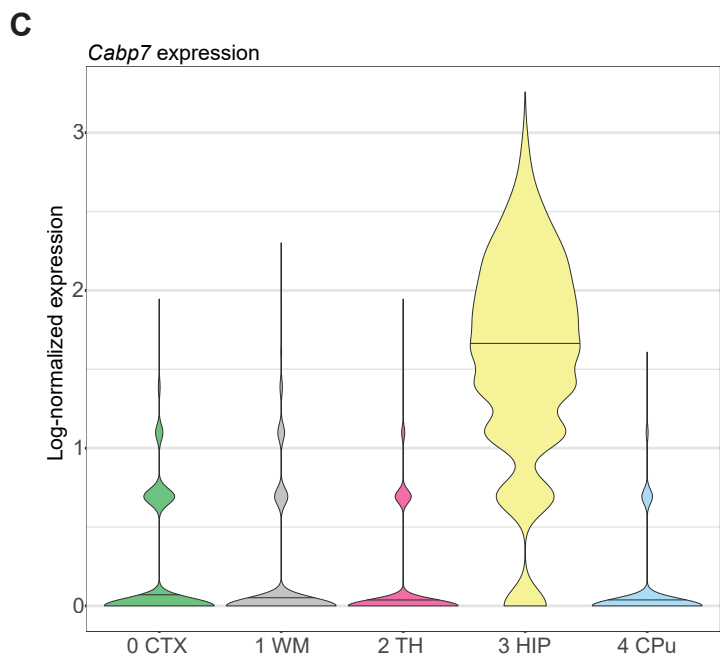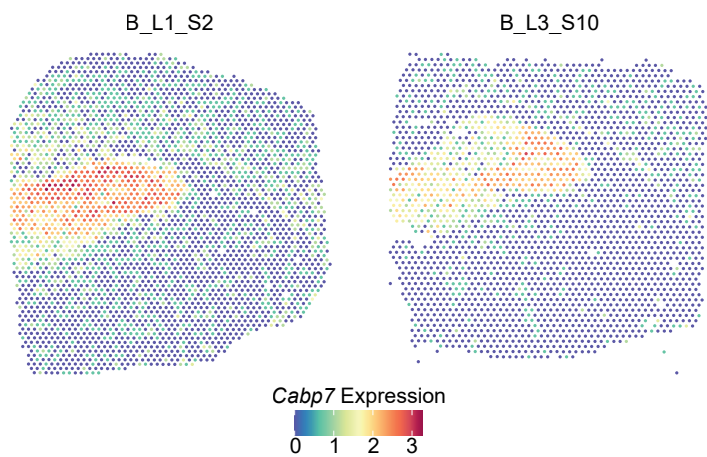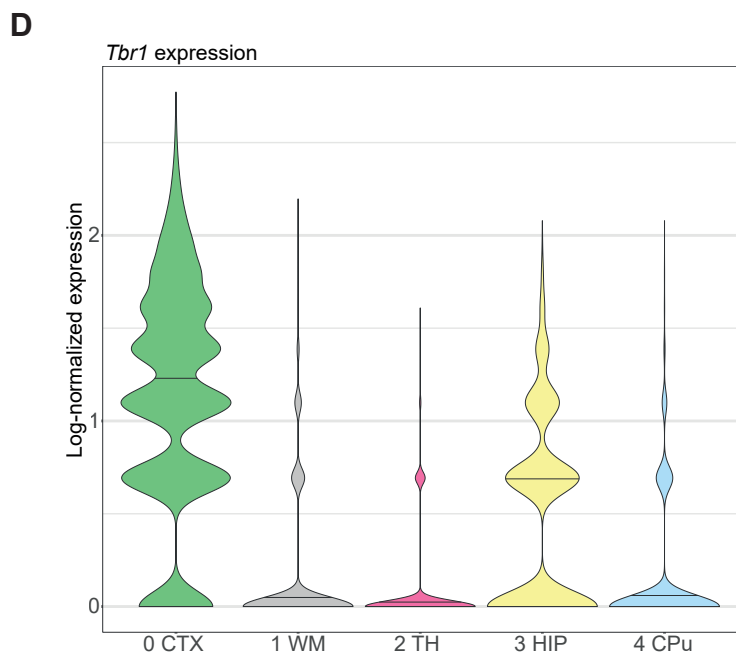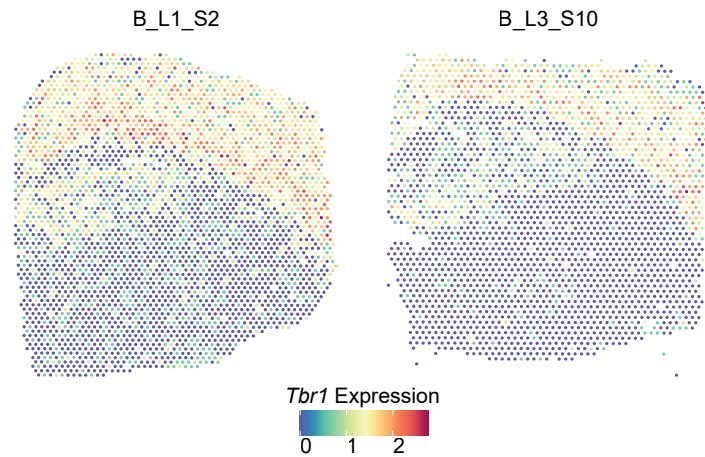

Supplementary Figure 2

Supplement: Supplementary file 2 — Supplementary Material 2: Supplementary Fig. 2. Whole clustering. A Four panels of UMAP representation of all spots passing quality control colored according to clusters, condition (treated or control), time points or sample of origin (from top to bottom and from left to right). The mean silhouette score is indicated in the top of each plot. B Spatial plots of clusters on tissue slices of samples at the D5 time point (treated samples on top, control samples on bottom). C and D Example of hippocampal and cortical marker (Cabp7 and Tbr1, respectively) expression levels after log2 transformation. Violin plots per cluster (top), spatial plots on the tissue slice of the control samples at the D5 time point (bottom). [file 40478_2026_2224_MOESM2_ESM.pdf]

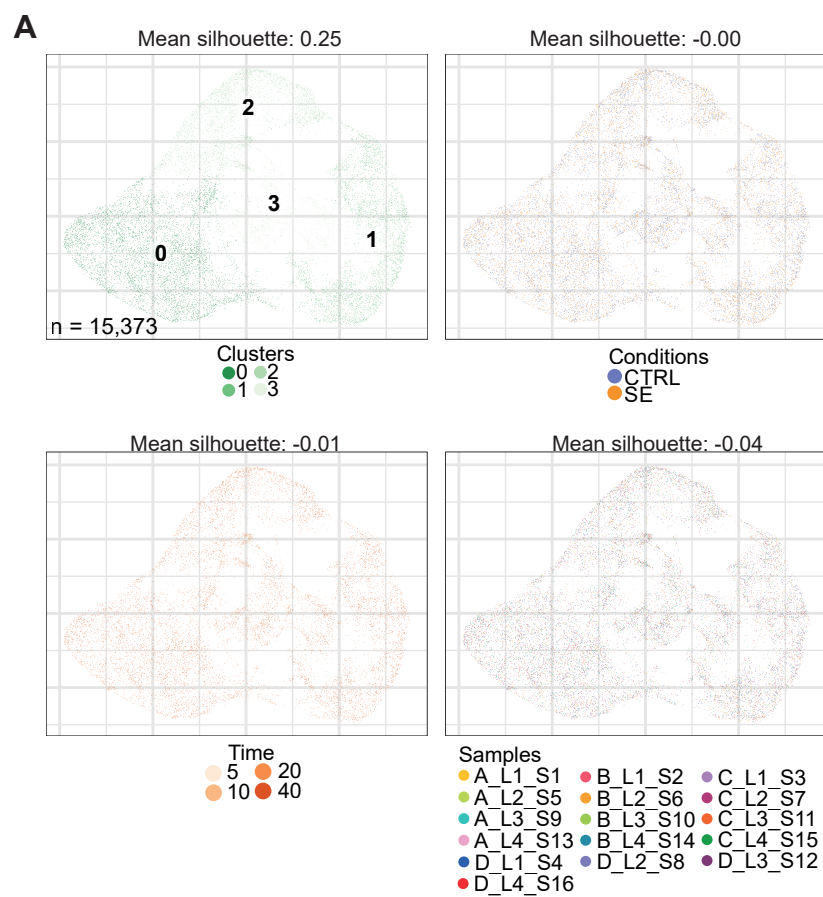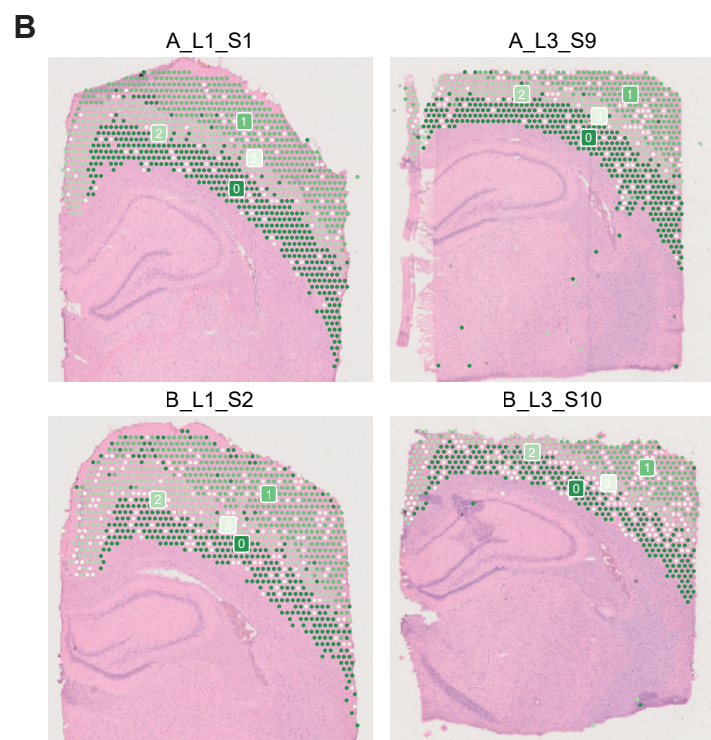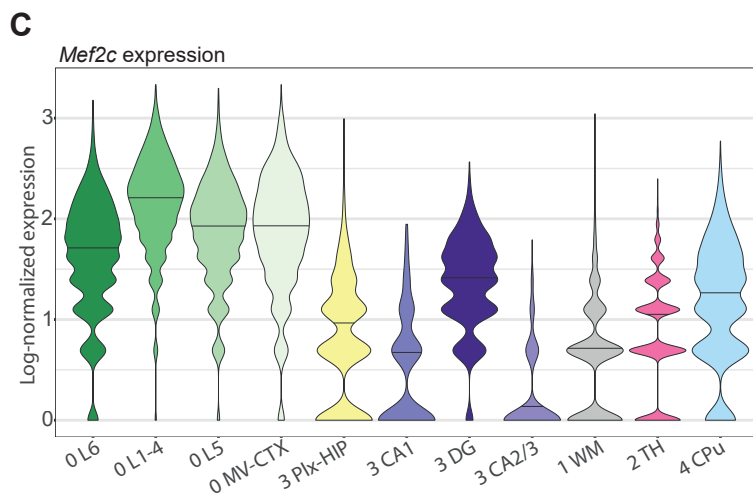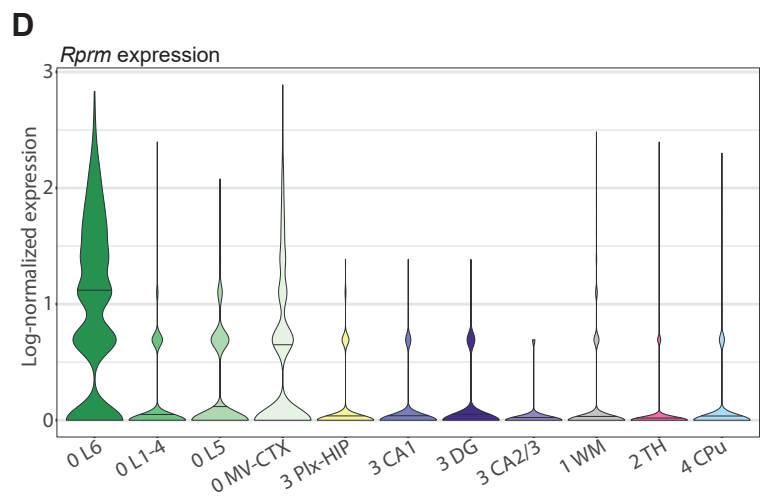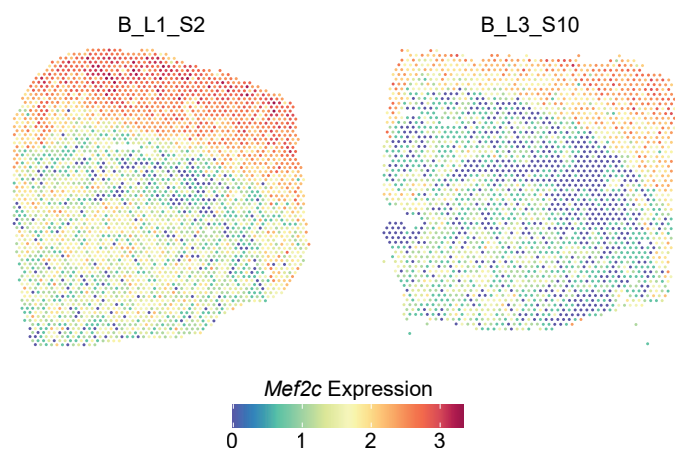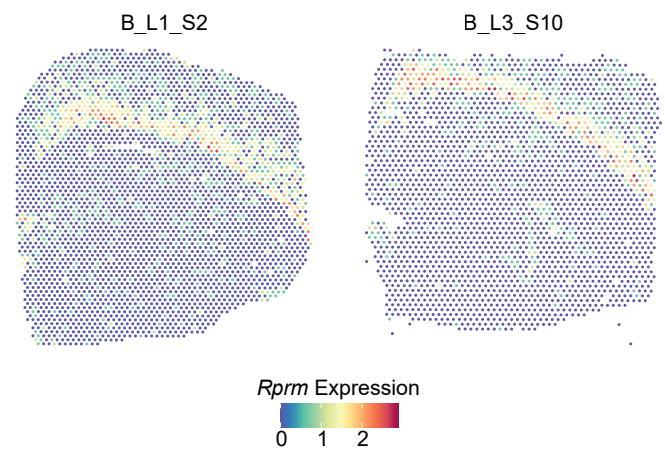

Supplementary Figure 3

Supplement: Supplementary file 3 — Supplementary Material 3: Supplementary Fig. 3. Sub-clustering of the cortex. A Four panels of UMAP representation of cortical cluster spots colored according to subcluster, condition (treated or control), time points or sample of origin (from top to bottom and from left to right). The mean silhouette score is indicated in the top of each plot. B Spatial plots of sub-clusters on tissue slices of samples at the D5 time point (treated samples on top, control samples on bottom). C and D Example of cortical layers 6a and 5 marker (Mef2c and Rprm) expression levels after log2 transformation. Violin plots per cluster (top), spatial plots on the tissue slice of the control samples at the D5 time point (bottom). [file 40478_2026_2224_MOESM3_ESM.pdf]

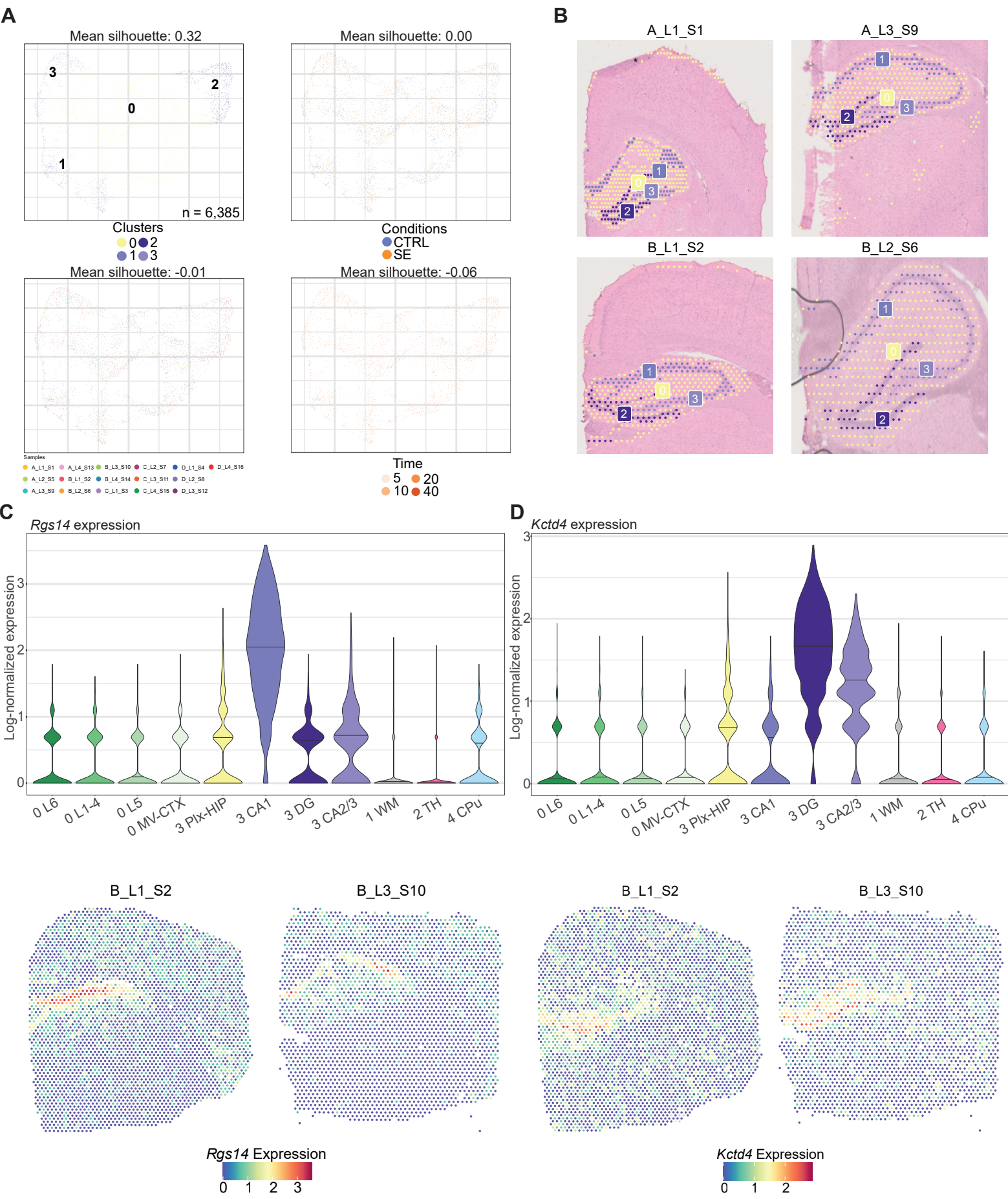

Supplementary Figure 4

Supplement: Supplementary file 4 — Supplementary Material 4: Supplementary Fig. 4. Sub-clustering of the hippocampus. A Four panels of UMAP representation of hippocampal cluster spots colored according to subcluster, condition (treated or control), time points or sample of origin (from top to bottom and from left to right). The mean silhouette score is indicated in the top of each plot. B Spatial plots of sub-clusters on tissue slices of samples at the D5 time point (treated samples on top, control samples on bottom). C and D Example of CA1 and DG marker (Rgs14 and Kctd4) expression levels after log2 transformation. Violin plots per cluster (top), spatial plots on the tissue slice of the control samples at the D5 time point (bottom). [file 40478_2026_2224_MOESM4_ESM.pdf]

**A**

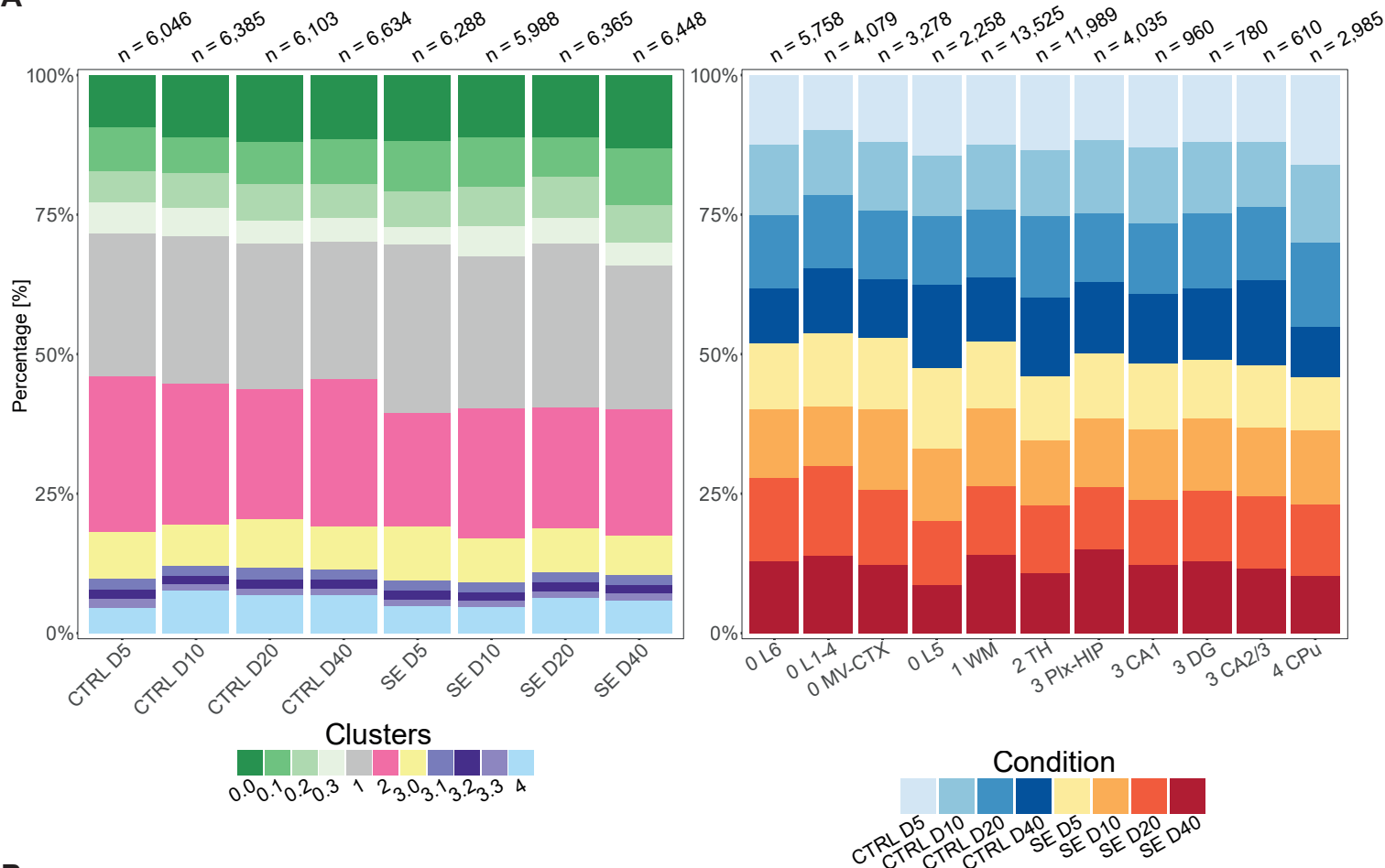

**B**

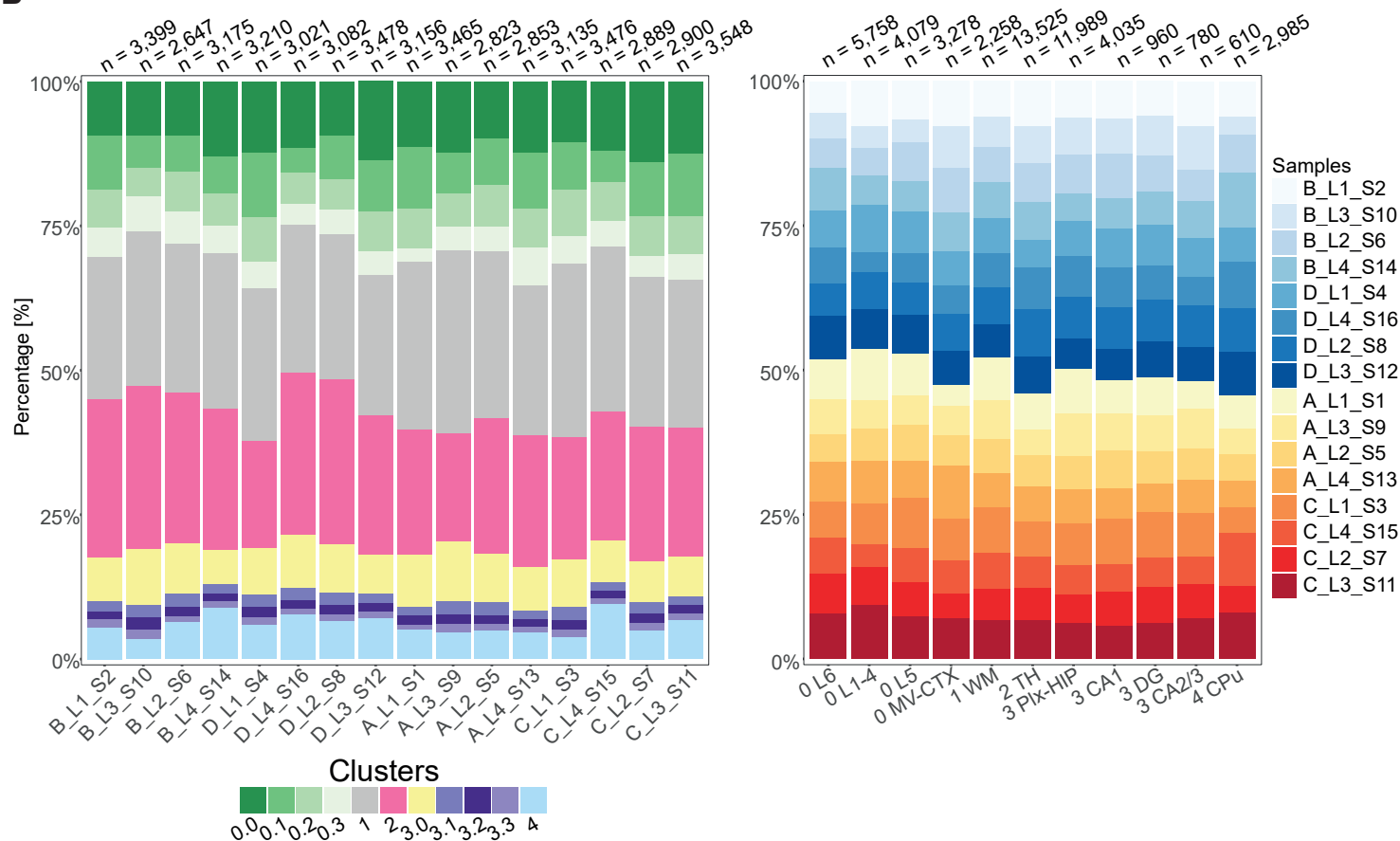

Supplementary Figure 5

Supplement: Supplementary file 5 — Supplementary Material 5: Supplementary Fig. 5. Visualisation of conditions and samples repartition by clusters. A Conditions repartition by clusters. On the left, the repartition of the different clusters for each condition and time, with the number of spots for each condition on the top, and on the right, the repartition of the different conditions and times for each cluster, with the number of spots for each cluster on the top. B Samples repartition by clusters. On the left, the repartition of the different clusters for each sample, with the number of spots for each sample on the top, and on the right, the repartition of the different samples for each cluster, with the number of spots for each cluster on the top. [file 40478_2026_2224_MOESM5_ESM.pdf]

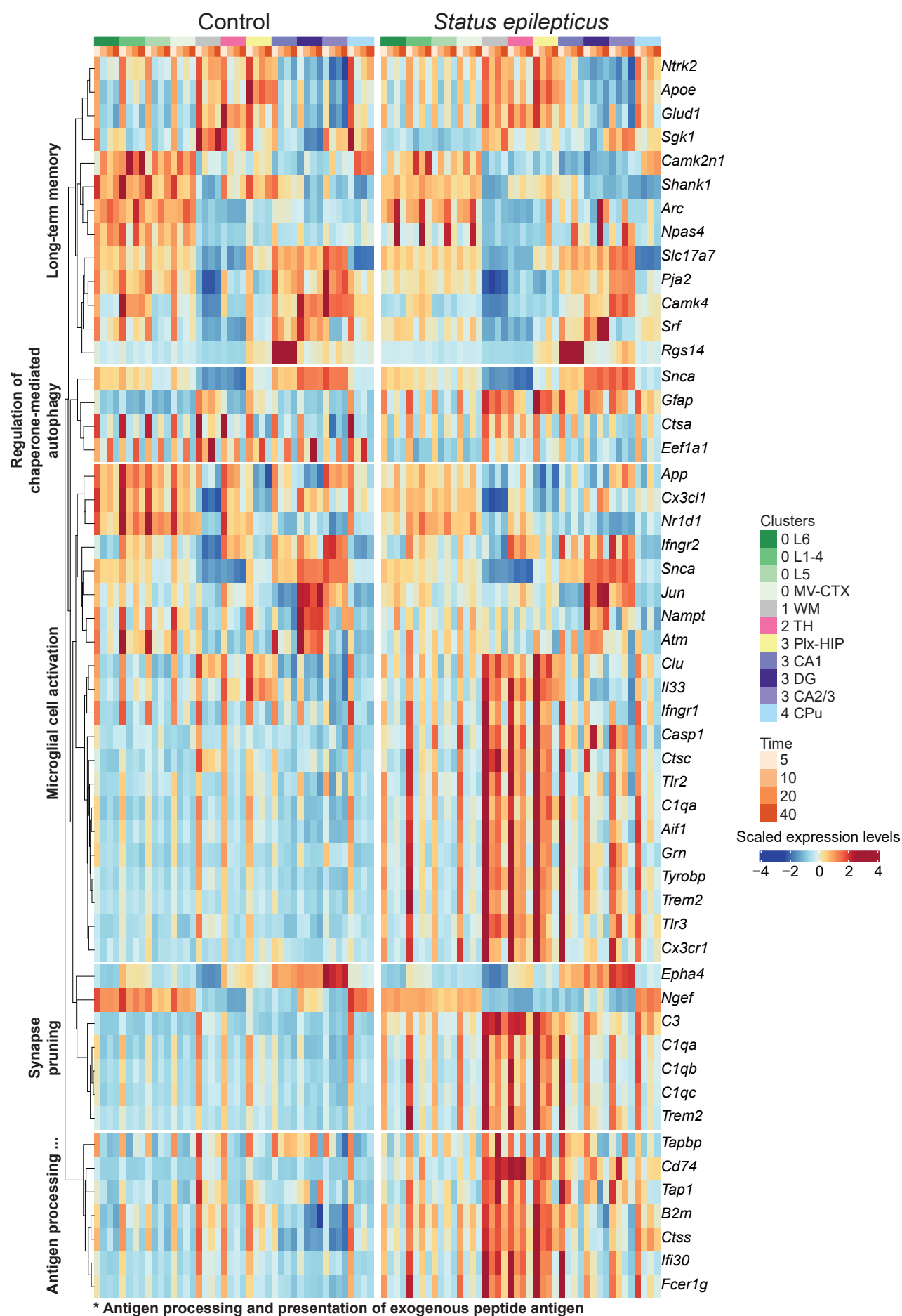

Supplementary Figure 6

Supplement: Supplementary file 6 — Supplementary Material 6: Supplementary Fig. 6. Heatmap of SE dysregulated genes grouped by cluster membership and GO:BP annotation. Heatmap showing the average expression levels of SE dysregulated genes associated with the following GO Biological Process terms: Antigen processing and presentation of exogenous peptide antigen, Long term memory, Regulation of chaperone-mediated autophagy, Microglial cell activation and Synapse pruning. Expression values are grouped by condition, cluster membership and time point. The SE dysregulated genes correspond to genes identified as differentially expressed between SE and CTRL conditions. [file 40478_2026_2224_MOESM6_ESM.pdf]

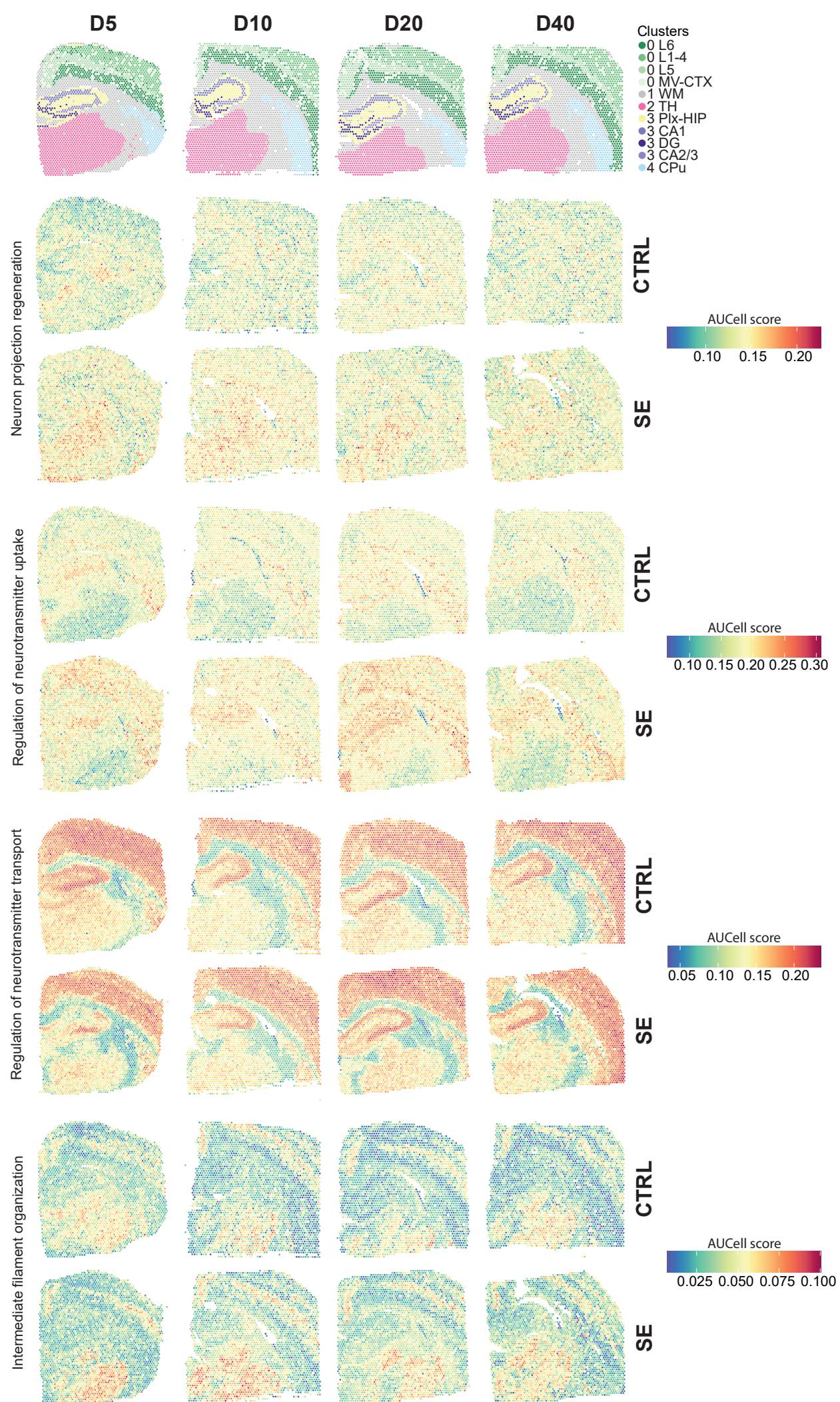

Supplementary Figure 7

Supplement: Supplementary file 7 — Supplementary Material 7: Supplementary Fig. 7. Spatial pathway activity. Visualisation of pathway activity calculated using AUCell on selected GO:BP terms a Neuron projection regeneration b Regulation of neurotransmitter uptake c Regulation of neurotransmitter transport d Intermediate filament organisation. [file 40478_2026_2224_MOESM7_ESM.pdf]

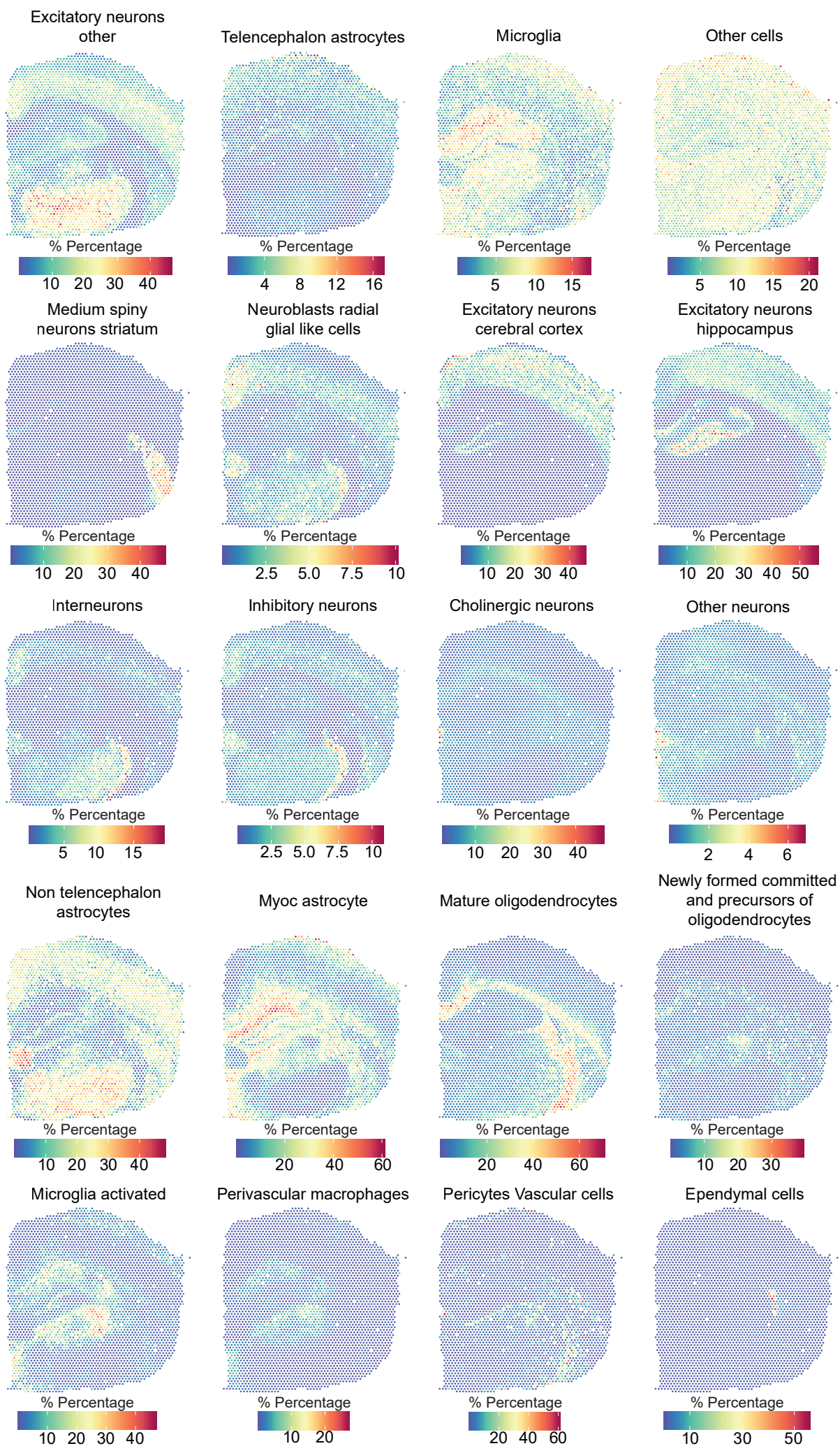

Supplementary Figure 8

Supplement: Supplementary file 8 — Supplementary Material 8: Supplementary Fig. 8. Spatial deconvolution at D5 after SE. Visualisation of the percentage of cell types per spot for the selected cell types on the Visium section A_L1_S1. [file 40478_2026_2224_MOESM8_ESM.pdf]

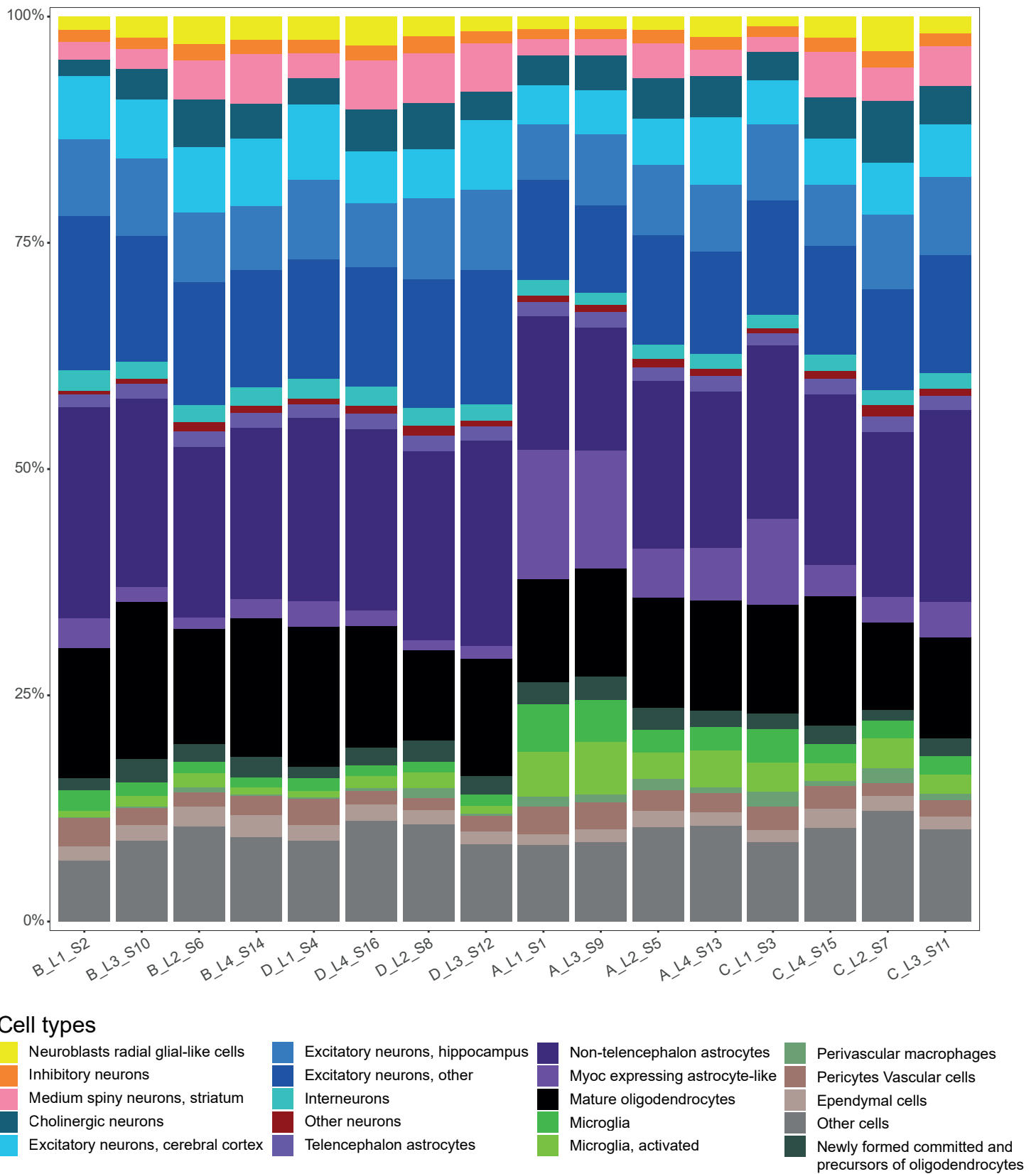

Supplementary Figure 9

Supplement: Supplementary file 9 — Supplementary Material 9: Supplementary Fig. 9. Cell-type composition by section. Barplots representing the cumulative estimated proportions of cell-types for all spots of each section. [file 40478_2026_2224_MOESM9_ESM.pdf]
