# Supplementary material for: Spatiotemporal transcriptomic mapping reveals region-specific glial activation and astrocyte shifts in epileptogenesis beyond the hippocampus
Source: Acta Neuropathol Commun. 2026 Jan 15;14:38. doi: 10.1186/s40478-026-02224-y (PMC12892793; doi:10.1186/s40478-026-02224-y)
Supplement: Supplementary file 10 — Supplementary Material 10: Supplementary Table 1. Sample metadata. Table summarizing the characteristics of brain slides used in this study, including: samples, stages, conditions, slides, numbers of spots. [file 40478_2026_2224_MOESM10_ESM.docx]

| Samples | Stage | Condition | Slides | SpatialFeaturePlot | Numbers of spots |
| --- | --- | --- | --- | --- | --- |
| A_L1_S1 | D5 | SE | L1 | Yes | 3,465 |
| A_L2_S5 | D10 | SE | L2 | No | 2,853 |
| C_L1_S3 | D20 | SE | L1 | Yes | 3,476 |
| C_L2_S7 | D40 | SE | L2 | No | 2,900 |
| B_L1_S2 | D5 | CTRL | L1 | Yes | 3,399 |
| B_L2_S6 | D10 | CTRL | L2 | No | 3,175 |
| D_L1_S4 | D20 | CTRL | L1 | Yes | 3,021 |
| D_L2_S8 | D40 | CTRL | L2 | No | 3,478 |
| A_L3_S9 | D5 | SE | L3 | No | 2,823 |
| A_L4_S13 | D10 | SE | L4 | Yes | 3,135 |
| C_L4_S15 | D20 | SE | L4 | No | 2,889 |
| C_L3_S11 | D40 | SE | L3 | Yes | 3,548 |
| B_L3_S10 | D5 | CTRL | L3 | No | 2,647 |
| B_L4_S14 | D10 | CTRL | L4 | Yes | 3,210 |
| D_L4_S16 | D20 | CTRL | L4 | No | 3,082 |
| D_L3_S12 | D40 | CTRL | L3 | Yes | 3,156 |
